# Arylsulfatase B induces melanoma apoptosis by the ubiquitin ligase COP1

**DOI:** 10.1016/j.jbc.2025.110402

**Published:** 2025-06-23

**Authors:** Sumit Bhattacharyya, Insug O-Sullivan, Herbert E. Whiteley, Jiyuan Yang, Fuming Zhang, Joanne K. Tobacman

**Affiliations:** 1 Research Service, Jesse Brown VAMC, Chicago, Illinois, USA; 2 Department of Medicine, University of Illinois Chicago, Chicago, Illinois, USA; 3 Veterinary Diagnostic Laboratory, College of Veterinary Medicine, University of Illinois Urbana-Champaign, Urbana, Illinois, USA; 4 Center for Biotechnology and Interdisciplinary Studies, Rensselaer Polytechnic Institute, Troy, New York, USA

**Keywords:** melanoma, chondroitin sulfate, N-acetylgalactosamine-4-sulfatase, sulfotransferase

## Abstract

Previous experiments in the syngeneic, murine, and subcutaneous model of malignant melanoma and human melanoma cells showed that treatment by recombinant human (rh) Arylsulfatase B (ARSB; N-acetylgalactosamine-4-sulfatase) markedly reduced the volume of tumors, improved survival, and inhibited invasiveness. In this report, the impact of ARSB on the progression of metastatic, pulmonary B16F10 melanomas in C57BL/6J mice is addressed, and the underlying apoptotic mechanism by which ARSB inhibits melanoma growth is identified. Exogenous ARSB, which has mannose 6-phosphate attachments, acts through insulin-like growth factor 2 receptor (IGF2R), a cation-independent mannose-6-phosphate receptor, and increases expression of constitutive photomorphogenic protein (COP)1. Expression of COP1, an E3 ubiquitin ligase, is increased by a decline in phospho(Ser473)-AKT1 and an increase in nuclear FOXO3a. ARSB-induced declines in carbohydrate sulfotransferase (CHST)15 expression and in transmembrane receptor tyrosine kinase-like orphan receptor 1 (ROR1) activation mediate the decline in phospho(Ser473)-AKT1. Inverse effects of rhARSB and ARSB knockdown on phospho(Ser473)-AKT1 indicate that ARSB acts as a tumor suppressor and that a decline in ARSB is pro-oncogenic. COP1, which inhibits ultraviolet-B stimulated growth in plants, suppresses nuclear ETS1 and ETS1-mediated expression of BCL2 in the murine melanomas and in human melanoma cells. These effects increase cytoplasmic cytochrome c, caspase-3/7 activation, and apoptosis. Since UVB exposure is recognized as a significant etiological factor in melanoma, identification of COP1 as an inhibitor of melanoma growth suggests the underlying presence of an ARSB-initiated growth inhibitory mechanism, analogous to that in plants, which contributes to the regulation of melanoma progression.

Globally, 510,000 occurrences of melanoma and 96,000 deaths are anticipated by 2040 (1, 2), even with the availability of immune checkpoint inhibitor treatment. Ultraviolet B (UVB) radiation has been identified as the main risk factor for melanoma occurrence (3, 4). In this report, we identify an increase in constitutive photomorphogenic 1 (COP1; RFWD2) as the mediator of increased apoptosis and inhibition of metastatic pulmonary melanomas in the syngeneic B16F10 murine model of metastatic melanoma. COP1, an E3 ubiquitin ligase, has been identified as a repressor of UVB-stimulated responses in plants and human cells (5, 6, 7, 8, 9, 10, 11, 12) but not previously considered in relation to melanoma apoptosis.

In this report, we present the signaling and transcriptional events initiated by arylsulfatase B (ARSB; N-acetylgalactosamine-4-sulfatase), which lead to COP1-mediated apoptosis of melanoma cells. In plants, COP1 interacts with UVB-stimulated UVR (UV Receptor) 8 and negatively regulates light signaling by inhibiting UVR8-induced downstream growth signaling by HY5 (elongated hypocotyl 5) (5, 6, 9, 10, 11, 12). In mammalian cells, COP1 ubiquitinates p53 and c-Jun as well as members of the ETS (E26 transformation-specific) family of transcription factors (13, 14, 15, 16, 17, 18, 19). Decline in nuclear ETS1 reduces BCL2 expression, leading to caspase activation (18, 19, 20, 21, 22).

In previous reports, we have detailed the inhibitory effects of exogenous, rhARSB on PD-L1 expression (23), invasiveness, and matrix metalloproteinase activity and expression in melanoma cell lines (24), and on improved survival and inhibition of growth of subcutaneous melanomas in the B16F10 cutaneous mouse model (25). Previous investigations indicated that changes in ARSB activity affected multiple signaling and transcriptional events, often by altered binding of galectin-3 or of the ubiquitous non-receptor tyrosine phosphatase SHP2 with more or less sulfated chondroitin 4-sulfate (C4S), following either a decline or an increase in ARSB (26, 27, 28, 29, 30, 31, 32). Decline in ARSB was associated with malignancy in mammary, colon, and prostate cells and tissues as well as in melanoma. Since the only established effect of ARSB is to hydrolyze the 4-sulfate group of C4S and DS, the effects of ARSB on signaling mechanisms and transcriptional events in these varied contexts are attributable to altered binding of critical molecules, such as galectin-3 or SHP2, with C4S.

For this report, we performed disaccharide analysis of malignant human melanoma cells following treatment by exogenous ARSB and by ARSB siRNA and considered these findings about the measured effects of ARSB on chondroitin 4-sulfate and on carbohydrate sulfotransferase (CHST) expression. These data enable consideration of how ARSB-induced changes in chondroitin 4-sulfation may impact the distribution and abundance of other sulfated glycosaminoglycans, particularly chondroitin sulfate E (CS-E; chondroitin-4,6-sulfate). Multiple reports have detected an association between an increase in CS-E and CHST15, which adds 6-sulfate groups to C4S to make CS-E, and malignancy (32, 33, 34, 35, 36, 37, 38).

In this report, we address how treatment by recombinant human ARSB impacts on apoptosis of melanoma by COP1 and identify a pathway by which ARSB-induced changes in chondroitin sulfation regulate COP1 expression. These interactions suggest how modifying chondroitin sulfation can regulate activation of critical signaling pathways and affect ubiquitination through the expression of COP1, thereby leading to inhibition of melanoma growth.

## Results

### ARSB treatment inhibits B16F10 metastatic pulmonary melanomas

**Figure 1:**
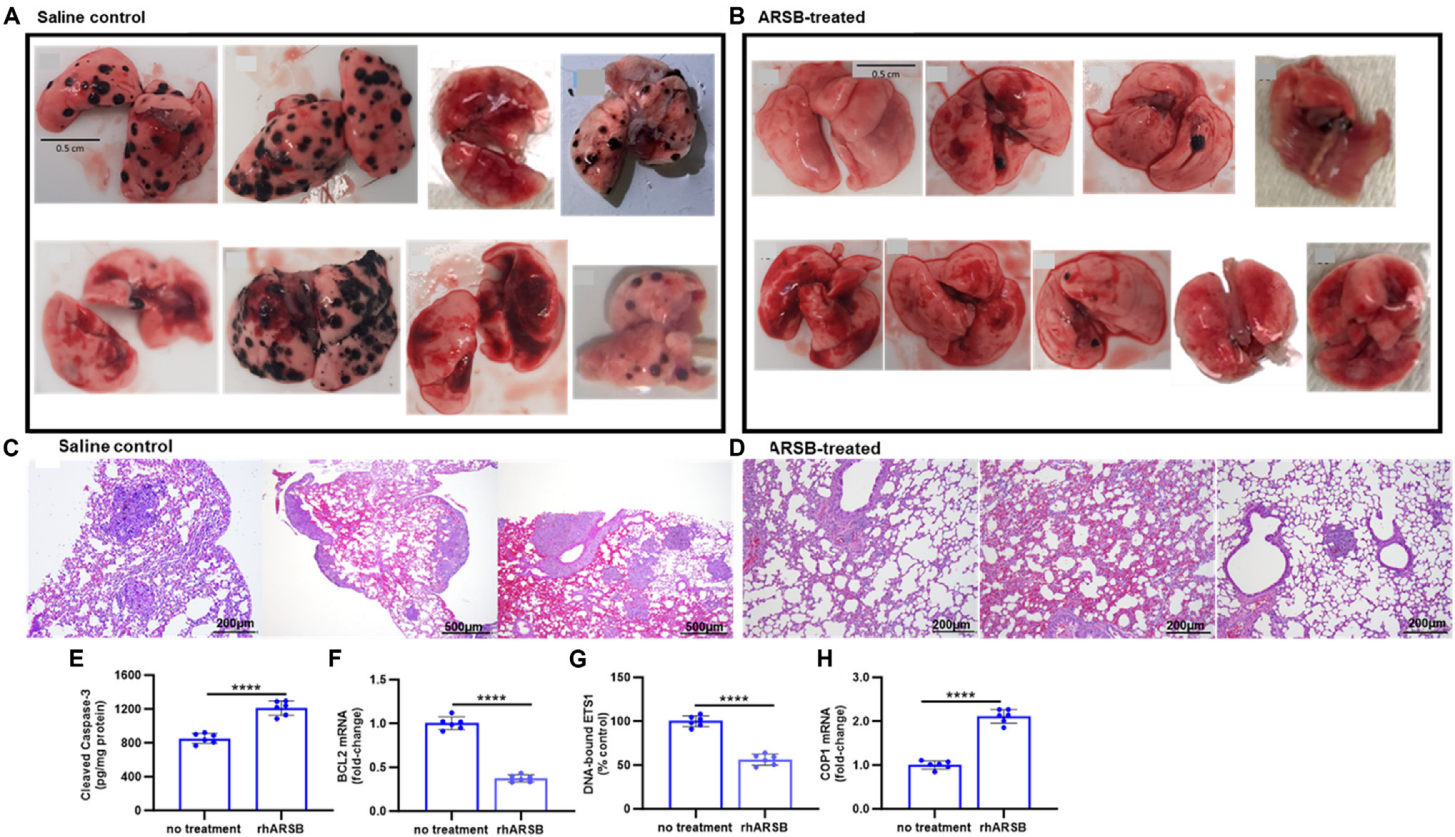
**Exogenous ARSB inhibits B16F10 pulmonary melanoma metastases**. *A*, images of lungs from 8 saline-treated, control pulmonary melanomas from C57BL/6J mice euthanized on day 14 following intravenous inoculation with 200,000 B16F10 mouse melanoma cells. Bar is 0.5 cm. *B*, images of lungs from 9 C57BL/6J mice euthanized on day 14 following intravenous inoculation with 200,000 B16F10 melanoma cells and treated with intravenous recombinant human ARSB (2.0 mg/kg) on days 2 and 7 following inoculation. Bar is 0.5 cm. *C* and *D*, histopathology demonstrates larger and more abundant melanomas in representative images of the untreated lung tissue, compared to the ARSB-treated tissue. Bar is 500 μm or 200 μm, as noted. In the lung melanomas, cleaved caspase-3 was increased by over 40% following rhARSB-treatment, compared to saline-treated control. *E*, cleaved caspase-3 was over 40% greater following rhARSB in the pulmonary melanomas. *F*, BCL2 mRNA expression was *lower* in the rhARSB-treated pulmonary melanomas. *G*, DNA-bound ETS1 declined in the rhARSB-treated pulmonary melanomas. *H*, an increase in caspase-mediated apoptosis was attributed to increased expression of the E3 ubiquitin ligase COP1 (constitutive photomorphogenic-1; RFWD2), leading to a decline in DNA-bound ETS1 and BCL2 expression in the lung tissue.

B16F10 melanoma cells were injected into the tail vein of C57BL/6J mice (n = 17), and nine mice were treated with rhARSB 0.2 mg/kg IV on Days 2 and 7 following tumor cell injection. Mice were euthanized on day 14, and photographs were taken of the lungs following excision and prior to allocation for further processing. Images of saline-treated controls (n = 8) (Fig. 1*A*) and rhARSB-treated mice (n = 9) (Fig. 1*B*)-show a marked increase in the number of metastatic nodules in the untreated mice, compared to the ARSB-treated mice. Representative histopathology demonstrates the greater abundance and size of metastatic nodules in the untreated (Fig. 1*C*), compared to the treated (Fig. 1*D*) mouse lungs.

### ARSB induces COP1-mediated apoptosis in melanomas

To explain the marked inhibition of melanoma proliferation, we considered whether exogenous ARSB could increase apoptosis of the melanomas. Cleaved caspase-3 was increased by ∼42% in the B16F10 lung melanomas (Fig. 1*E*). Apoptosis was associated with a decline in BCL2 (Fig. 1*F*) and attributed to a decline in DNA-bound ETS1 (Fig. 1*G*), which is associated with effects on BCL2 expression (20, 21, 22). Since COP1 ubiquitinates transcription factor ETS1 (15, 16, 17, 18, 19), expression of the E3 ubiquitin ligase constitutive photomorphogenic (COP)-1 was assessed in the lung melanomas (Fig. 1*H*) and shown to increase following rhARSB treatment. Similar findings were identified in the subcutaneous melanoma tissues (Supplementary Information 1), in which cleaved caspase-3 was increased by ∼51%. The mice with subcutaneous B16F10 melanomas were previously reported to have increased survival and smaller tumors following rhARSB (25). Findings indicate that rhARSB suppression of melanoma growth in both the metastatic pulmonary melanomas and the subcutaneous melanomas is attributable to enhanced apoptosis.

### Exogenous ARSB induces COP1-mediated apoptosis in A375 melanoma cells

**Figure 2:**
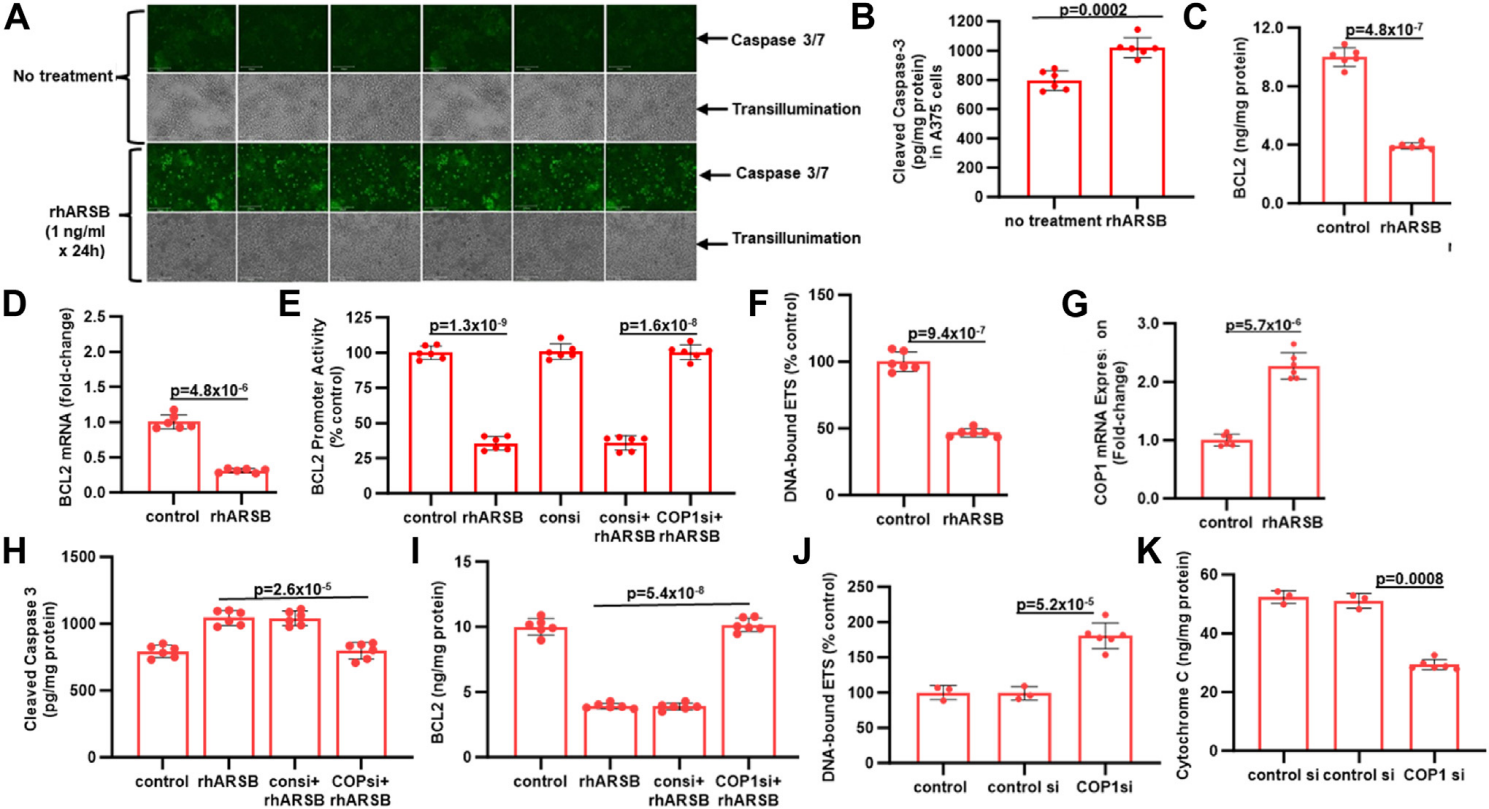
**Exogenous ARSB induces apoptosis in A375 melanoma cells and normal human melanocytes by COP1-mediated effects**. *A*, when cultured A375 cells were exposed to rhARSB (1 ng/ml) x 24 h and exposed to fluorescent caspase 3/7 dye, marked increase in caspase 3/7 was evident by fluorescent microscopy compared with untreated cells. *B*, measurement of cleaved caspase 3 in the A375 melanoma cells confirms increase of about 30% in cleaved caspase 3 following exposure to rhARSB. *C*, following rhARSB, BCL2 protein declined in the A375 cells. *D*, BCL2 mRNA declined about 70% in the A375 cell treated with rhARSB. *E*, BCL2 promoter activity decreased following rhARSB and the decline was inhibited by COP1 silencing. *F*, DNA-bound ETS1 was reduced following rhARSB. *G*, exogenous ARSB increased COP1 mRNA expression by about 2.6-fold. *H*, the rhARSB-induced increase in cleaved caspase-3 was reversed by COP1 knockdown. *I*, BCL2 protein declined following rhARSB and COP1 knockdown reversed the inhibition. *J*, COP1 knockdown increased DNA-bound ETS1, consistent with the COP1 association with decline in DNA-bound ETS1. *K*, cytoplasmic cytochrome C was reduced by COP1 knockdown in the A375 cells.

Live-cell imaging was performed of cultured human A375 melanoma cells treated with rhARSB (1 ng/ml x 24h) and then exposed to caspase 3/7 fluorescent dye x 24h. Images show a marked increase in cleaved caspase-3 following rhARSB, compared to untreated controls (Fig. 2*A*). Increased cleaved caspase-3 was measured in cultured A375 cells following rhARSB (1 ng/ml x 24h) (Fig. 2*B*) and increased by ∼28% over untreated controls. BCL2 protein (Fig. 2*C*), mRNA (Fig. 2*D*), and promoter activity (Fig. 2*E*) were reduced by rhARSB. DNA-bound ETS1 (Fig. 2*F*) declined, and COP1 expression (Fig. 2*G*) increased following rhARSB.

Consistent with the requirement for COP1 for apoptosis, COP1 siRNA blocked the observed increase in cleaved caspase-3 (Fig. 2*H*) and the decline in BCL2 protein (Fig. 2*I*). When COP1 was silenced by siRNA, DNA-bound ETS1 (Fig. 2*J*) increased and cytoplasmic cytochrome C (Fig. 2*K*) declined significantly. Similar findings were detected in normal human melanocytes and murine B16F10 melanoma cells (Supplementary Information 2). These results indicate that the apoptotic pathway involving COP1 is also present in normal cells and is enhanced by rhARSB.

### Chondroitin sulfate and disaccharide composition of pulmonary melanomas and A375 cells following ARSB treatment

To address how the known function of ARSB, which is to remove the 4-sulfate group from N-acetylgalactosamine 4-sulfate residues at the non-reducing end of chondroitin 4-sulfate (C4S) or dermatan sulfate, could lead to enhanced apoptosis in melanoma, the effects of rhARSB on C4S and associated parameters were determined in the B16F10 melanomas and A375 cells. Total sulfated glycosaminoglycans, C4S, carbohydrate sulfotransferase (CHST) 11, which adds 4-sulfate group to unsulfated N-acetylgalactosamine residues, CHST15, which adds 6-sulfate group to C4S, and chondroitin disaccharides were assessed.

**Figure 3:**
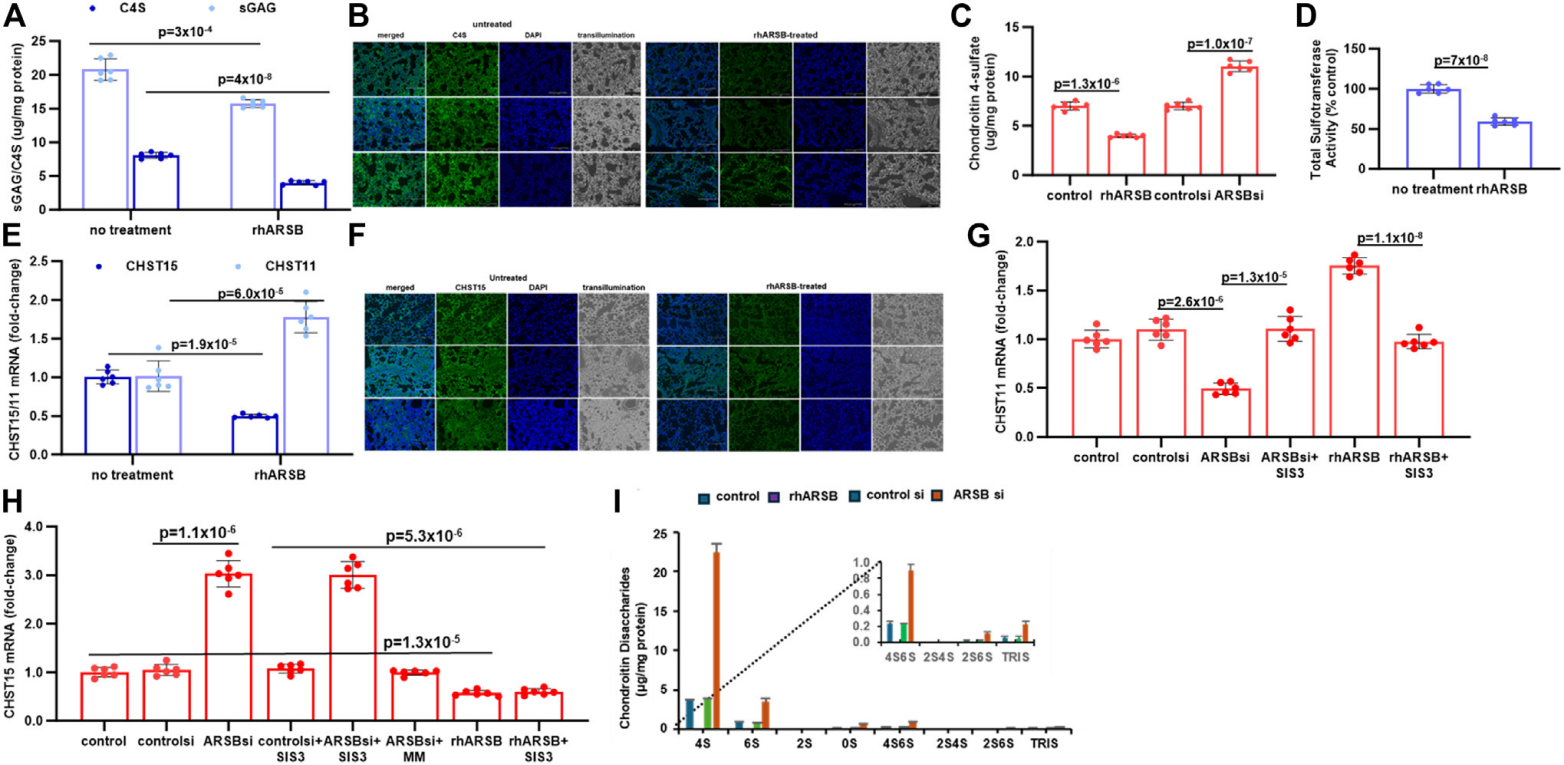
**Impact of rhARSB on sulfated glycosaminoglycans, chondroitin sulfotransferases, and chondroitin disaccharides**. *A*, in the B16F10 lung melanomas, total sulfated glycosaminoglycans (sGAG) and chondroitin 4-sulfate (C4S) were measured by the dimethylmethylene blue assay and for C4S following immunoprecipitation by specific C4S antibody. They were significantly reduced by rhARSB treatment. *B*, chondroitin 4-sulfate (C4S) fluorescent immunostaining of ARSB-treated and control pulmonary melanoma tissue confirms the marked decline in C4S following rhARSB treatment. Bar = 150 μm. DAPI-stained nuclei (*blue*) and *green* fluorescent C4S are compared. *C*, in the A375 cells, C4S content was reduced by rhARSB and increased when ARSB was silenced. *D*, total sulfotransferase activity declined in the pulmonary melanoma tissue following rhARSB treatment. *E*, mRNA expression of CHST (carbohydrate sulfotransferase) 15 was reduced in the lung melanomas following exogenous ARSB. In contrast, the CHST11 expression was significantly increased. *F*. representative images of CHST15 immunofluorescence confirm decline in CHST15 protein in the ARSB-treated lung tissue, compared to the untreated control. Bar = 150 μm. DAPI-stained nuclei are *blue* and FITC-stained CHST15 are *green*. G, in A375 cells, CHST11 expression is inhibited by SIS3, a specific inhibitor of Smad3. ARSB silencing reduces CHST11 expression, and rhARSB increases CHST11 expression. *H*, in contrast, CHST15 mRNA expression is not inhibited by SIS3, but is inhibited by mithramycin (MM). This is consistent with a transcriptional mechanism requiring galectin-3 and Sp1 (Supplementary Information 5 and References 23 and 25). *I* and *J*, disaccharide analysis shows marked increase in 4S disaccharides following ARSB silencing, in contrast to decline following treatment by rhARSB. This suggests that the hydrolysis of the 4-sulfate group by rhARSB exceeds the capacity of CHST11 to increase the C4S content. ARSB knockdown increases the 4-sulfate disaccharide unit to over 22 μg/mg protein. 4S6S disaccharides, as present in chondroitin sulfate E and synthesized by CHST15, increase following ARSB knockdown, but are present at a concentration of <1 μg/mg protein.

In the pulmonary B16F10 melanomas, total sulfated GAGs and C4S declined following rhARSB (Fig. 3*A*). C4S content declined from 8.05 ± 0.45 μg/mg protein to 4.02 ± 0.28 μg/mg protein following ARSB treatment. C4S immunostaining of lung tissue confirmed the decline in C4S following ARSB treatment, compared to saline-treated control (Fig. 3*B*). In the B16F10 subcutaneous melanomas, we reported previously that ARSB-treated mice had marked declines in total sulfated glycosaminoglycans (GAGs) and chondroitin 4-sulfate (C4S), as measured by the dimethylmethylene blue (DMMB) assay (25). Similarly, in the A375 cells, C4S content declined by ∼36% (Fig. 3*C*). Since the chondroitin sulfate content results from both degradation and production of chondroitin sulfate, sulfotransferase activity and expression of the carbohydrate sulfotransferases CHST11 (adds 4-sulfate to unsulfated chondroitin) and CHST15 (adds 6-sulfate to C4S) were determined in the pulmonary melanoma tissues. Total sulfotransferase activity declined over 40% (Fig. 3*D*), CHST11 expression increased, and CHST15 expression declined in the lung tissues following exogenous ARSB (Fig. 3*E*). Imaging confirms the decline in CHST15 expression in the lung melanomas (Fig. 3*F*). This is similar to what was previously reported in the B16F10 cutaneous melanomas following exogenous ARSB (25). In A375 cells, ARSB silencing reduced CHST11 expression (Fig. 3*G*) and increased CHST15 expression (Fig. 3*H*). In normal melanocytes and B16F10 melanoma cells, similar effects of ARSB treatment on CHST15 and CHST11 expression were detected (Supplementary Information 3).

Different transcription factors regulate the expression of CHST11 and CHST15 in the melanoma cells. The rhARSB-induced increase in CHST11 mRNA was inhibited by the SMAD3 blocker SIS3 (Fig. 3*G*). In contrast, CHST15 expression was not inhibited by SIS3, but was blocked by mithramycin, an inhibitor of Sp1-mediated transcription (Fig. 3*G*). Specific mechanisms involving phospho-SMAD3 and Sp1 are presented (Supplementary Information 3 and (25)).

### Disaccharide analysis following rhARSB and ARSB siRNA in A375 cells

Disaccharide analysis is the gold standard for identification of specific chondroitin sulfations present in cells or tissues and provides essential information about the distribution of disaccharides following cell treatments. A375 melanoma cells were exposed to exogenous ARSB (1 ng/ml x 24h) or silenced by ARSB siRNA; duplicate samples were analyzed for total content of chondroitin sulfates (CS), heparan sulfates (HS), and hyaluronan (HA). Analysis showed markedly greater abundance of CS in the control samples (mean = 7.76 μg), compared to either HS (mean = 101.5 ng) or HA (mean = 17.5 ng) (Supplementary Information 4*A*). Following ARSB silencing, the total CS increased 7.7-fold to almost 60 μg. Treatment by rhARSB reduced the total CS to 1.5% of the baseline control value.

Specific disaccharide analysis was performed to detect the content of 8 chondroitin sulfate disaccharides, including 0S, 2S, 4S, 6S, 2S4S, 2S6S, 4S6S, and TriS (S = sulfate). Targeted mass spectrometry showed the distribution of the specific disaccharides in the A375 cells following treatment by ARSB siRNA or exogenous ARSB. The percentage of 4S disaccharides of total disaccharides increased by ∼7.5% when ARSB was silenced. The 4S disaccharides declined by almost 11% following rhARSB, reflecting the effect of ARSB to remove 4-sulfate groups (Supplementary Information 4*B*). Supplementary Information 4*C* presents the concentration of each disaccharide as ng/ml. 4S disaccharides increased to ∼48,000 ng/ml when ARSB was silenced and declined to ∼73 ng/ml following rhARSB, from baseline value of ∼5682 ng/ml. The doubly sulfated 4S6S disaccharides, which define chondroitin sulfate E, declined following rhARSB and increased following ARSB siRNA, consistent with the observed changes in CHST15 expression. The 4S6S disaccharides declined to 0.9 ng/ml following rhARSB (Supplementary Information 4*C*). In contrast, ARSB silencing increased 4S6S disaccharides to ∼1940 ng/ml from baseline of 403 ng/ml.

Using values of protein in mg/ml determined from replicate samples, the 4S disaccharide concentration increased to ∼22.41 μg/mg protein following ARSB siRNA and declined to ∼0.06 μg/mg protein after exogenous ARSB (Fig. 3, *I* and *J*) (Supplementary Information 4*D*). 4S6S was negligible after rhARSB and increased to ∼0.90 μg/mg protein following ARSB silencing. These findings are consistent with the decline in CHST15 expression following rhARSB and the increase following ARSB silencing. The decline in C4S disaccharides following rhARSB indicates that the sulfatase effect of rhARSB exceeds the impact of increased expression of CHST11 to increase C4S.

### Impact of CHST15 silencing on mediators of apoptosis

**Figure 4:**
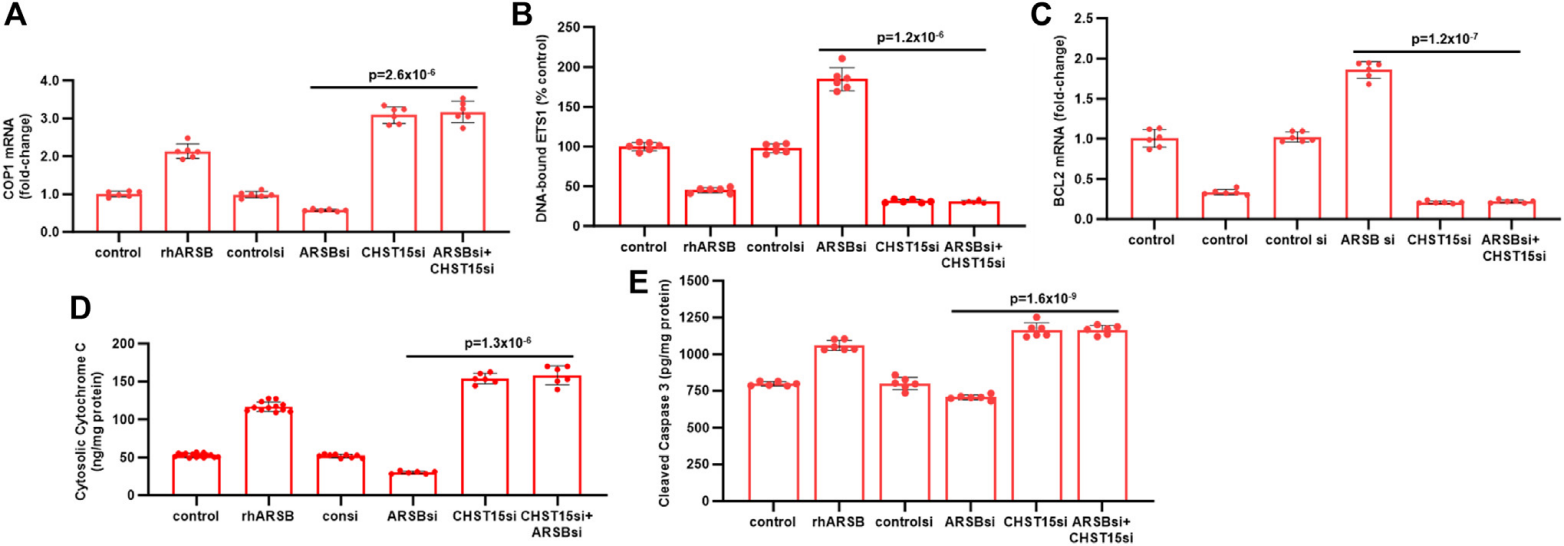
**Impact of CHST15 silencing on apoptotic pathway**. *A*, COP1 expression increased following rhARSB or CHST15 siRNA. ARSB siRNA reduced COP1 expression, and this decline was reversed by CHST15 siRNA. *B*, in A375 cells, DNA-bound ETS1 declined significantly following rhARSB and increased when ARSB was silenced. Knockdown of CHST15 also inhibited the DNA-bound ETS1 and reversed the effect of ARSB silencing. *C*, mRNA expression of anti-apoptotic BCL2 increased when ARSB was silenced, and. CHST15 siRNA inhibited the ARSB siRNA-induced increase in BCL2 expression. *D*, cytosolic Cytochrome C, a marker of mitochondrial disruption and apoptosis, increased following rhARSB or CHST15 siRNA. CHST15 siRNA reversed the ARSB silencing-induced decline in Cytosolic Cytochrome C. *E*, cleaved caspase 3 was measured by ELISA and, increased following rhARSB or CHST15 silencing. ARSB knockdown reduced cleaved caspase-3 and CHST15 siRNA reversed this decline.

CHST15 and chondroitin sulfate E disaccharides were reduced following exogenous ARSB, suggesting that a decline in CHST15 might contribute to rhARSB-induced apoptosis. In A375 cells, CHST15 knockdown inhibited the effects of ARSB siRNA on COP1 (Fig. 4*A*), DNA-bound ETS1 (Fig. 4*B*), BCL2 mRNA (Fig. 4*C*), cytosolic cytochrome C (Fig. 4*D*), and cleaved caspase-3 (Fig. 4*D*). Effects of CHST15 silencing are pro-apoptotic, in the same direction, and even more pronounced than the effects of rhARSB.

### Exogenous ARSB and CHST15 knockdown increase COP1 by phospho(Ser473)-AKT1/FOXO3

**Figure 5:**
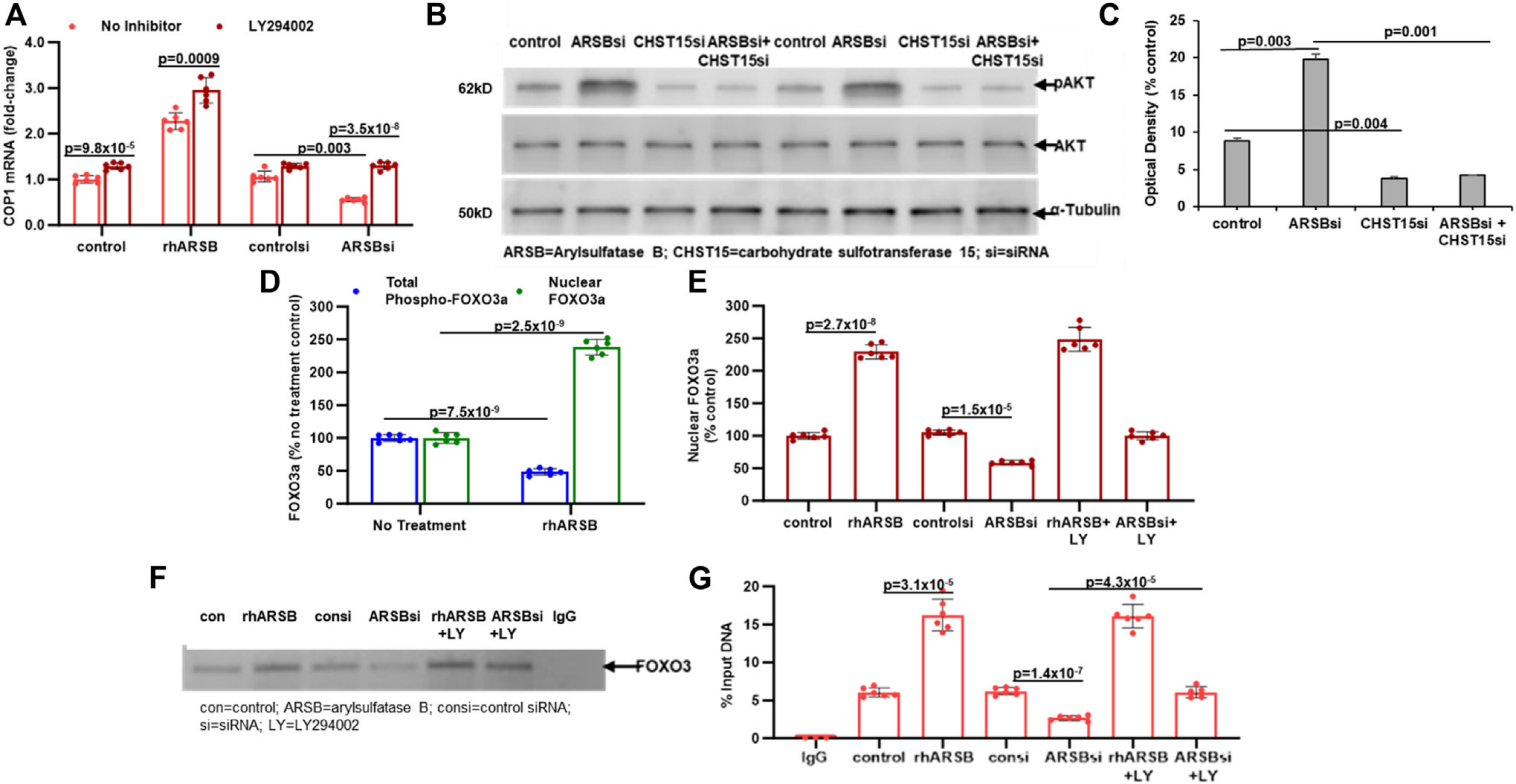
**Phospho-AKT1 inhibition leads to FOXO3-mediated increase in COP1 expression**. *A*, the PI3K inhibitor LY294002 significantly increased COP1 mRNA in control, rhARSB, control siRNA, and ARSB siRNA A375 samples. *B*, Western blot with duplicate independent samples showed that the ARSB siRNA-induced increase in phospho(Ser473)-AKT1 was inhibited by CHST15 siRNA and that CHST15 siRNA markedly reduced phospho(Ser473)-AKT in the A375 cells. Pan-AKT1 was unchanged. *C*, measurements of optical density support the observed effects of ARSB and CHST15 knockdown on phospho(Ser473)-AKT1. *D*, in the pulmonary melanoma tissue, rhARSB reduced total phospho-FOXO3a and increased nuclear FOXO3a to 2.5 times the baseline level. *E*, the PI3K/pAKT1 inhibitor LY294002 (LY) did not inhibit the rhARSB-induced increase in nuclear FOXO3a and slightly increased the ARSB siRNA-induced decline in nuclear FOXO3a in the A375 cells. *F*, chromatin immunoprecipitation detected the extent of binding of FOXO3a with the COP1 promoter and showed increased binding following rhARSB and decline following ARSB siRNA. The PI3K inhibitor LY294002 (LY) inhibited the ARSB siRNA-induced decline in binding to the promoter. *G*, % input DNA increased following rhARSB and declined following ARSB silencing. LY294002 inhibited the effect of ARSB siRNA.

The mechanism by which exogenous ARSB increased COP1 expression was investigated by testing the effect of potential inhibitors, including knockdown of the mannose 6-phosphate receptor IGF2R by siRNA (Supplementary Information 5). IGF2R siRNA blocked the rhARSB-induced increase in COP1 expression, but IGF2R siRNA had no independent impact on COP1 expression (Supplementary Information 6*B*). Neither galectin-3 siRNA nor SHP2 inhibition by PHPS1 reversed ARSB siRNA-induced suppression of COP1 (data not shown). Several inhibitors of cell signaling, including p38-MAPK inhibitor SB203580, c-Jun peptide inhibitor, which blocks JNK, Rho/Rac1 GTPase inhibitor (NSC23766), and STAT3 inhibitor niclosamide, had no impact on the ARSB siRNA-induced decline in COP1 expression (data not shown). In contrast, inhibition of PI3K/AKT by LY294002 (LY) increased COP1 in control, rhARSB-treated, ARSB-silenced, and control siRNA A375 cells (Fig. 5*A*). Western blot showed that phospho(Ser473)-AKT1 increased following ARSB silencing and declined following CHST15 silencing (Fig. 5*B*). (Supplementary Information 7). The combination of ARSB and CHST15 silencing blocked the ARSB siRNA-induced increase (Fig. 5*B*). Optical density measurements confirm the visual impression (Fig. 5*C*).

Phospho(Ser473)-AKT1 has been linked to nuclear translocation and activation of FOXO3 in melanocytes and melanoma (39, 40, 41, 42, 43, 44, 45, 46, 47). In the A375 cells, total phospho-FOXO3a declined following rhARSB, but nuclear FOXO3a increased (Fig. 5*D*). The PI3K/pAKT1 inhibitor LY294002 increased the ARSB siRNA-induced decline in nuclear FOXO3a (Fig. 3*E*). Chromatin immunoprecipitation assay indicated FOXO3a binding to the COP1 promoter. Exogenous ARSB and exogenous ARSB in combination with LY294002 increased binding, compared to untreated control. ARSB siRNA reduced binding, which was increased by the combination of ARSB siRNA and LY294002 (Fig. 5*F*). Measurements of optical density and input DNA are consistent with the visual impression of the blot (Fig. 5*G*). These results indicate that COP1 expression is inversely related to PI3K/pS473-AKT1 and that CHST15 knockdown impacts on PI3K/pS473-AKT1 to reduce COP1 expression.

### Inhibition of Phospho-Tyr-ROR1 by rhARSB, CHST15 siRNA, and Cirmtuzumab reduces phospho(S473)-AKT1 and mediates apoptosis

CHST15 and its product chondroitin sulfate E are associated with several signaling pathways affecting oncogenesis (32, 33, 34, 35, 36, 37, 38). The receptor tyrosine kinase-like orphan receptor 1 (ROR1) was known to stimulate PI3K/pAKT activation (48, 49, 50, 51, 52) and was previously reported to be associated with reduced invasive effects following CHST15 knockdown in malignant mammary cells (38). This mechanism was evaluated as a potential pathway by which CHST15 inhibition might suppress phospho(Ser473)-AKT1 activation in the melanoma cells.

**Figure 6:**
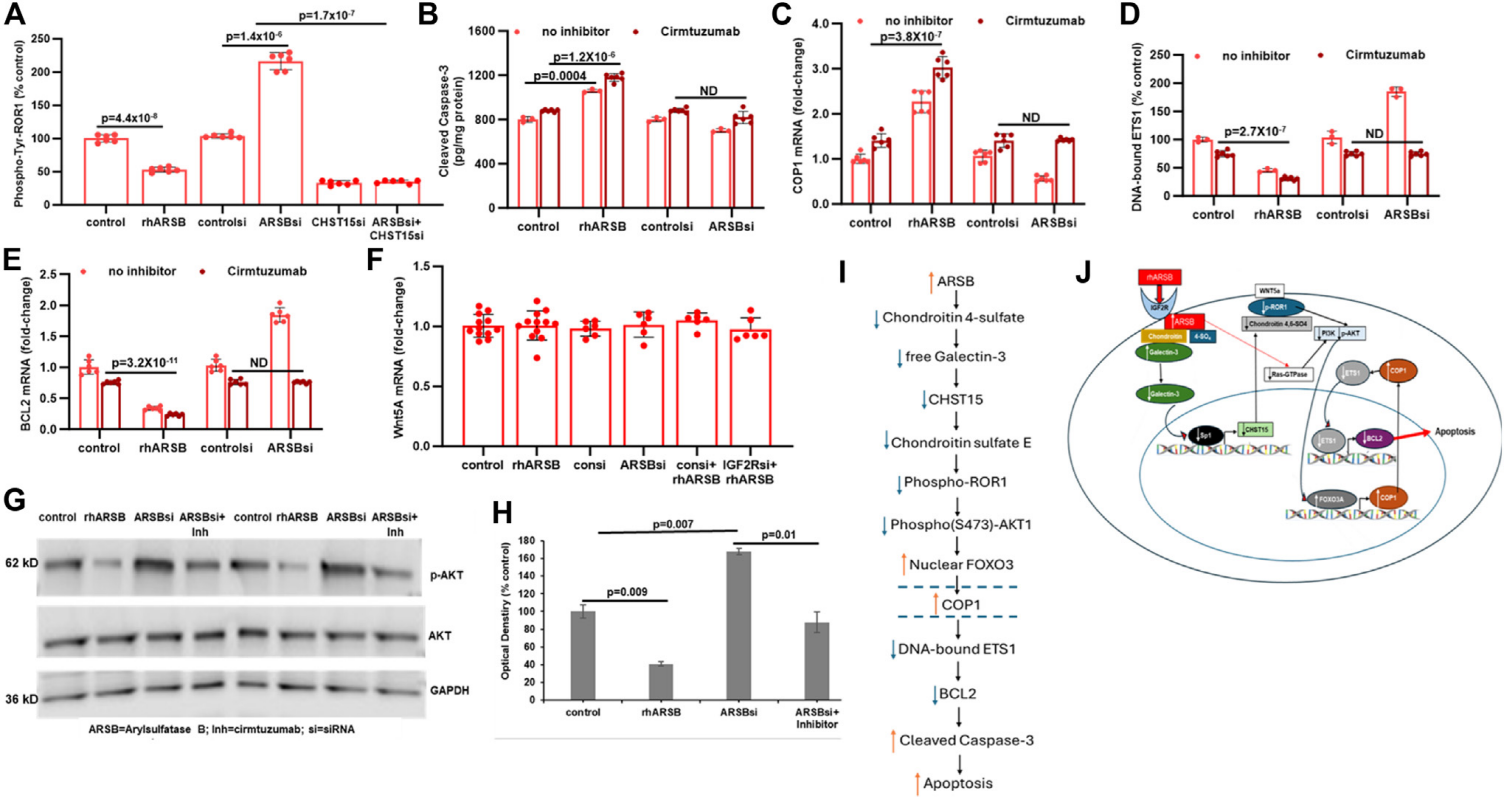
**Inhibition of phospho(Tyr)-ROR1 by rhARSB, CHST15 siRNA, or Cirmtuzumab increases COP1 expression and apoptosis by effects on phospho(Ser473)-AKT1**. *A*, phospho-(Tyr)-ROR1 was reduced by rhARSB and CHST15 siRNA and was increased by ARSB silencing. CHST15 silencing inhibited the ARSB siRNA-induced increase. *B*, the monoclonal antibody inhibitor of phospho(Tyr)-ROR1, cirmtuzumab, increased the effects of control, rhARSB, control siRNA, and ARSB siRNA on cleaved caspase 3. *C*, phospho(Tyr)-ROR1 inhibition by cirmtuzumab increased COP1 expression in A375 cells. *D* and *E*, DNA-bound ETS1 and BCL2 mRNA declined following cirmtuzumab. *F*, mRNA expression of Wnt5A, a known activator of phospho(Tyr)-ROR1, was unaffected by rhARSB or ARSB siRNA in the A375 cells. *G*, Western blot of duplicate, independent samples showed decline in phospho(Ser473)-AKT1 bands following rhARSB. The ARSB siRNA-induced increase in band intensity was reduced by cirmtuzumab (Inh), indicating that inhibition of phospho(Tyr)-ROR1 reduced phospho(Ser473)-AKT1 activation. *H*, densitometry of phospho(Ser473)-AKT1 bands confirms the observed decline by rhARSB, increase by ARSB siRNA, and inhibition of ARSB siRNA by the phospho(Tyr)-ROR1 inhibitor. *I*, overall signaling and transcriptional events are shown in the pathway schematic, noting that COP1 is critical for rhARSB-induced apoptosis and is the product of an rhARSB-initiated transcriptional pathway. *J*, schematic shows the impact of rhARSB and ARSB-mediated effects on transcriptional events involving CHST15, BCL2, FOXO3a, and COP1.

We tested the effects of rhARSB, ARSB siRNA, CHST15 siRNA, and the combination of ARSB and CHST15 siRNAs on phospho(Tyr)-ROR1 in the A375 cells (Fig. 6*A*). Exogenous ARSB and CHST15 knockdown reduced p-ROR1, and the ARSB siRNA-induced increase was inhibited by CHST15 siRNA. Cirmtuzumab, a humanized monoclonal antibody which targets p-ROR1 (53, 54, 55, 56), increased cleaved caspase-3 (Fig. 6*B*) and COP1 mRNA (Fig. 6*C*), and reduced DNA-bound ETS1 (Fig. 6*D*) and BCL2 mRNA (Fig. 6*E*). These effects suggest that a decline in phospho-ROR1 activation following rhARSB, CHST15 siRNA, or Cirmtuzumab provides a pathway that coordinates a tyrosine-kinase-like surface receptor with chondroitin sulfate and melanoma apoptosis. Since Wnt5A interacts with ROR1 (57), we confirmed that A375 cells express Wnt5A (Fig. 6*F*) and that Wnt5A is unaffected by changes in ARSB.

Western blot showed that treatment with the p-ROR1 antagonist Cirmtuzumab reduced the ARSB siRNA-induced increase in phospho(S473)-AKT1, without change in total AKT1 (Fig. 6*G*) (Supplementary Information 8). Optical densities confirmed the visual impression (Fig. 6*H*). Schematic representation summarizes the signaling and transcriptional events which follow treatment by rhARSB (Fig. 6, *I* and *J*). The pathway includes COP1-induced decline in DNA-bound ETS1, leading to decline in BCL2 expression and increased cleaved caspase-3 and enhanced apoptosis. In addition to the COP1-mediated apoptotic pathway, the rhARSB-induced changes in chondroitin sulfates and in CHST15 expression lead to a p-ROR1/p-AKT1/FOXO3-mediated pathway by which COP1 expression is increased.

## Discussion

The novel pathways identified in the current studies integrate rhARSB-initiated effects on chondroitin sulfation with signaling events that lead to enhanced COP1 expression. Other pathways modified by ARSB-induced changes in chondroitin 4-sulfation have been previously reported (26). These include enhanced binding of galectin-3 to less sulfated chondroitin 4-sulfate (C4S) and increased binding of SHP2 to more highly sulfated C4S. SHP2, a ubiquitous non-receptor tyrosine phosphatase, regulates phosphorylation of phospho-ERK1/2 and phospho-p38 MAPK. Sustained phosphorylation of these critical signaling molecules, when SHP2 is bound with C4S and unavailable to remove phosphates from these kinases, influences vital signaling cascades in cells and tissues of diverse origins, as previously summarized (26).

The cellular uptake of rhARSB in the melanoma cells is mediated by insulin-like growth factor 2 receptor (IGF2R), the paternally imprinted, cation-independent mannose-6-phosphate receptor (58, 59, 60) (Supplementary Information 3). IGF2R is not a signaling receptor and has no kinase domain, but is associated with the uptake of lysosomal enzymes into intracellular endosomal compartments. The relationship between IGF2R and endosomal uptake provides an avenue for exogenous ARSB-initiated intracellular effects, as evident in the treatment of Mucopolysaccharidosis VI by replacement therapy with exogenous ARSB (58). IGF2 was shown to allosterically interfere with binding of two lysosomal enzymes to the IGF2R, suggesting that interaction/competition between ARSB and IGF2 may affect cellular responsiveness to exogenous ARSB (59). In protooncogenic cells, upregulation of IGF2 was associated with evasion of apoptosis, and IGF2 overexpression was associated with the development of colorectal carcinomas and activation of pAKT signaling (60). The current findings show that IGF2R silencing blocked the effects of rhARSB in normal melanocytes and A375 cells. Intersections of IGF2-IGF2R with insulin and IGF1 and their receptors and with IGF-binding proteins provide avenues by which endogenous insulin biology and signaling responses may modify sensitivity to paracrine or exogenous ARSB and affect the endosomal uptake of ARSB. Interactions between ARSB and endogenous mediators of insulin and IGF1/2 signaling are of great interest, but beyond the scope of this report.

Enhanced apoptotic cell death is one of several mechanisms that may contribute to regulated cell death. Potentially, other mechanisms, including necroptosis, pyroptosis, and ferroptosis, may participate to some extent in the rhARSB-mediated process. The potential connectivity among these pathways has not been addressed in relation to sulfations or the availability of sulfate (61, 62). Treatment with rhARSB or by ARSB siRNA in A375 melanoma cells impacts cytokine production (unpublished data), suggesting that inflammatory mechanisms may also contribute to the rhARSB-induced enhancement of apoptosis and to other pathways of cell death. The impact of treatment by rhARSB on reduction of the number of pulmonary metastases (Fig. 1, *A* and *B*) and the previously reported decline in subcutaneous melanoma volume and increase in survival (25) may be attributable, in an unknown proportion, to mechanisms other than apoptosis. The data presented do not show specific cellular effects associated with apoptosis, such as chromatin condensation, plasma membrane blebbing, or chromatin condensation, focusing on elucidation of the BCL2-mediated biochemical pathway. Other data we have reported indicate that rhARSB reduces expression of matrix metalloproteinases and invasiveness (24), which certainly can contribute to the observed inhibition of tumor propagation by effects other than the direct effect on cell death. Also, the previously reported rhARSB-induced decline in PD-L1 expression due to epigenetic effects suggests that endogenous effects on checkpoint inhibition may also affect tumor aggressiveness (23) following rhARSB.

This report identifies several negative signaling events following exogenous ARSB, including declines in ETS1, BCL2 expression, CHST15 expression, phospho(Tyr)-ROR1 activation, and pS473-AKT1 activation. An increase in nuclear FOXO3 leads to increased COP1, which initiates the apoptotic cascade. Manipulation of ARSB and subsequent impact on chondroitin sulfation may make difficult targets, such as inhibition of pS473-AKT1, more amenable to sustained, effective intervention. The subsequent, enhanced FOXO3a nuclear effect on COP1 expression leads to apoptosis of the melanoma cells, consistent with the role of FOXO3a as a tumor suppressor (41).

Inverse to the impact of exogenous ARSB and pertinent to the development of melanoma, ARSB silencing increased CHST15 expression, abundance of C4,6S disaccharides, and activation of phospho(Tyr)-ROR1 and phospho(Ser473)-AKT1 and reduced nuclear FOXO3a and COP1 expression. Apoptosis of cells damaged by UV-induced mutations might escape destruction when COP1 expression is reduced following decline in ARSB. ARSB activity is responsive to external stimuli, including hypoxia and increased salt exposure (26, 27), which may contribute to a decline in apoptosis.

The experiments in this report indicate that exogenous, bioactive rhARSB inhibits melanoma progression by stimulating apoptosis through effects of the E3 ubiquitin ligase constitutive photomorphogenic 1 (COP1; RFWD2), a known inhibitor of growth response to ultraviolet B (UVB) light. In plants, COP1 combines with UVB-activated UV-B Receptor 8 (UVR8), one of the wavelength-specific responders to light signaling, which is crucial for proper growth. A COP1-UVR8 complex suppresses UVB activation of light signaling by inhibition of UVR8 binding with elongated hypocotyl 5 (HY5) or by direct ubiquitination of HY5 (5, 6, 9, 10, 11, 12). HY5, a bZIP-type transcription factor in multiple plant species, regulates the expression of over 3000 genes in response to light (11). By suppression of UVB activation of HY5, COP1 inhibits UVB-initiated growth in plants. In human epidermal keratinocytes, silencing COP1 increased UVB-mediated transcriptional effects and UVB reduced COP1 expression (7, 8), consistent with the inhibitory role of COP1 in melanoma detailed in this report, since human melanoma growth is stimulated by UVB exposure. Interestingly, BCL2 was reported to be downregulated following UV irradiation (63) and identified as a target of UV-inducible apoptosis (64). Also, ETS transcription factors have been associated with a UV damage signature and mutagenesis in melanoma (65). Disruption at ETS binding sites is attributed to GG doublet formation at the canonical 5′-GGAA-3′ ETS binding site (66). These associations between UV exposure, COP1, ETS1, BCL2, and melanoma apoptosis can provide new insight into fundamental melanoma pathophysiology, although no precise common mechanism between UVB exposure and decline in ARSB has been identified. Expression of other E3 ligases, including ITCH1, FBXW7, CUL4A, and SAG/RBX2/ROC2, was unchanged by rhARSB, suggesting a specific link between COP1 and chondroitin 4-sulfation.

In our efforts to determine how ARSB-induced changes in chondroitin sulfates links to melanoma apoptosis, disaccharide analysis provides an indirect method. Direct quantitative analysis of the sulfated GAGs remains a challenge, since the number of disaccharide units, the extent of sulfation, the sites of sulfation, variation in the arrangement of disaccharide units, and the impact of specific proteoglycans all contribute to their heterogeneity. However, addressing the 4-sulfation at the non-reducing end of C4S can provide some clarification, since binding attributable to the presence or absence of this specific sulfate group appears to affect the availability of other molecules, such as SHP2 and galectin-3, for other interactions. Disaccharide analysis provides overall information about the specific units present, and the effects of rhARSB and ARSB siRNA on the disaccharide distribution help to elucidate the overall pattern of chondroitin sulfations.

The broad and sustained impact of ARSB and associated changes in C4S on the binding of critical molecules with C4S and on subsequent phosphorylations and signaling can lead to transcriptional events and effects on biological processes, including ubiquitination, motility, and growth. These changes may be cumulative and irreversible. Whether exposure to exogenous ARSB can reverse the impact of decline in ARSB due to environmental exposures, such as hypoxia and high salt, and, thereby, normalize aberrant cellular processes or enhance apoptosis of damaged cells requires further investigation.

## Experimental procedures

### B16F10 metastatic melanomas in C57BL/6J mice

Eight-week-old female C57BL/6J mice (n = 17) were purchased (Jackson Laboratories) and housed in the Veterinary Medicine Unit at the Jesse Brown Veterans Affairs Medical Center (JBVAMC) (25). Principles of laboratory animal care were followed, and all procedures were approved by the AALAC-accredited Animal Care Committee of the JBVAMC. Mice were fed a standard diet and maintained in groups of three in a cage with routine light–dark cycles. Mice were ear-tagged for identification and divided into untreated and treated groups. To test the effectiveness of recombinant ARSB on pulmonary melanomas, B16F10 cells were cultured in DMEM (ATCC 30–2002) with 10% FBS under the recommended conditions. 200,000 B16F10 melanoma cells in saline were injected into the lateral tail vein of 17 14-week-old female C57BL/6J mice. Recombinant bioactive ARSB (R&D Systems) at a dose of 0.2 mg/kg BW was injected into the lateral tail vein of mice in the treated group on days 2 and 7 (n = 9). The untreated mice (n = 8) received parallel vehicle (saline) injections. The animals were euthanized by isoflurane inhalation and decapitation on day 14. At the time of euthanasia, lungs and other tissues were harvested. Pulmonary nodules were counted, and the lungs were photographed *en bloc*. Lung tissue was then divided into cassettes, which were placed in 10% formalin in preparation for paraffin blocks or frozen in liquid nitrogen for further processing.

### Cell culture of A375 melanoma cells and normal human melanocytes

A375 human melanoma cells (CRL-1619, ATCC) were grown in Dulbecco’s modified Eagle medium (DMEM) supplemented with 10% fetal bovine serum (FBS; ATCC) and 1x penicillin-streptomycin (ATCC). Cells were maintained at 37 °C in a humidified, 5% CO_2_ environment with media exchange every 2 days. Confluent cells in T-25 flasks were harvested using EDTA-trypsin (ATCC) and sub-cultured. Primary normal human adult epidermal melanocytes (PCS-200–013, ATCC) were cultured in dermal cell basal medium (PCS-200-030, ATCC) supplemented with adult melanocyte growth kit (PCS-200–042, ATCC). The cells were maintained at 37 °C in a humidified, 5% CO_2_ environment with media exchange every 3 days. Confluent cells in T-25 flasks were harvested by trypsin for primary cells (ATCC) and sub-cultured. B16F10 mouse melanoma cells were procured (ATCC), and cells were cultured in DMEM supplemented with 10% FBS and 1x penicillin-streptomycin. Cells were screened for pathogens by IDEXX BioAnalytics (Columbia, MO, USA). Certificates of Analysis for A375 cells, normal human melanocytes, and B16F19 cells confirmed the authenticity of the cell lines and their lack of *mycoplasma* contamination. Cells were maintained at 37 °C in a humidified, 5% CO_2_ environment with media exchange every 2 days, and confluent cells in T-25 or T-75 flasks were harvested by EDTA-trypsin and sub-cultured.

### Silencing of insulin-like growth factor 2 receptor (IGF2R), constitutive photomorphogenic (COP)1, carbohydrate sulfotransferase (CHST)15, galectin-3, and N-acetylgalactosamine-4-sulfatase (Arylsulfatase B, ARSB) by siRNA

The mRNA expression of IGF2R, COP1, CHST15, galectin-3, and ARSB was silenced by specific siRNA using standard procedures (30). Small-interfering RNA for IGF2R (Assay ID s7218, Thermo Fisher Scientific), COP1 (RFWD2; Assay ID s59691, Thermo Fisher), and CHST15 (Assay ID s28016, Thermo Fisher) were obtained and effectiveness was verified by QRT-PCR (Supplementary Information 4). Knockdown of ARSB and galectin-3 by siRNA was previously detailed (30). Briefly, the melanoma cells and normal melanocytes were grown to ∼70% confluence, then silenced by adding 0.6 μl of 20 μM siRNA (150 ng), mixed with 100 μl of serum-free medium and 12 μl of HiPerfect Transfection Reagent (Qiagen). Media were changed after 24h, and cell treatments were initiated.

### Treatment of A375 human melanoma and melanocytes cells by exogenous ARSB and other agents

Cells were treated by exogenous, bioactive rhARSB (1 ng/ml; R&D). Treatments were for 24h, unless indicated otherwise. Some cell preparations were treated with exogenous, bioactive rhGALNS (galactose 6-sulfate sulfatase) (1 ng/ml x 24 h; R&D #8269-SU). Cells were treated with several inhibitors, including: ERK activation inhibitor peptide 1, cell-permeable (10 μM; Sigma); p38α-MAPK inhibitor (10 μM; PH797804; Selleckchem, Houston, TX); a cell-permeable thiazolidinone compound which inhibits c-Myc-Max dimerization (64 μM, 10058-F4, Tocris Bioscience, Bio-Techne); p38 MAPK inhibitor SB203580 (10 μM; Tocris Bioscience); c-Jun peptide (400 μM; R&D Systems); Rho/Rac 1 inhibitor NSC23766 (1 ng/ml; Tocris Bioscience); PI3K inhibitor LY294002 (50 μM; Calbiochem, Millipore Sigma); STAT3 inhibitor Niclosamide (16 μM, Selleckchem), and Zilovertamab (50 μg/ml; UC-961; Cirmtuzumab, Medchemexpress).

### Total sulfotransferase activity

Total sulfotransferase activity was determined using the Universal Sulfotransferase Activity kit (R&D Systems) which detects activity of all sulfotransferases present. The reaction uses 3-phosphoadenosine-5-phosphosulfate (PAPS) as the sulfate donor and malachite green phosphate detection reaction to detect the phosphate released from PAPS by added inositol monophosphatase-3. The activity is normalized with total cellular protein and expressed as percentage of control.

### Measurements of phospho-proteins by ELISA

Phospho-ERK1(T202/Y204)/ERK2(T185/Y187) and Phospho-SHP2(Y542) in the tumor extracts were measured by specific DuoSet sandwich ELISAs kit (R&D Systems), as per manufacturer’s protocol. Concentrations of phospho-ERK1/2 or phospho-SHP2 in the samples were extrapolated from a curve derived using known standards. Phospho(T180/Y182)-p38 MAPK in the tumor extracts was measured in cell samples using a commercial Pathscan Sandwich ELISA (Cell Signaling Technology, Inc). Phospho(Tyrosine)-ROR1 in cell extracts was measured by a commercial sandwich ELISA (RayBiotech).

Phospho-FOXO3/FOXO3A (Ser253) cell-based phosphorylation ELISA kit (LS-F1512, LifeSpan BioSciences) was used to measure total cellular and nuclear-bound FOX03 in A375 cells and B16F10 mouse pulmonary melanomas. This is a sandwich ELISA kit for semi-quantitative measurements and uses an anti-pan FOXO3a antibody coated onto wells of a 96-well plate for binding. A375 cells were treated with rhARSB, ARSB siRNA, control siRNA, ARSB siRNA in combination with LY294002 and rhARSB in combination with LY294002 and either total cellular extract or nuclear extract was pipetted into wells of the coated plate and bound to the immobilized pan-FOXO3a antibody. B16F10 melanoma sample or nuclear extract of the melanoma tissue sample was added to other coated wells. Wells were washed and rabbit anti-phospho(S253)-FOXO3a antibody was added for detection of phosphorylated FOXO3a in the total cellular or tissue extract. In other wells, nuclear extracts from A375 melanoma cells or B16F10 lung melanomas were pipetted into wells coated with the pan-FOXO3a antibody and rabbit anti-FOXO3a antibody was added for detection. Unbound antibody was washed off, and HRP-conjugated anti-rabbit IgG or HRP streptavidin was pipetted into the wells. The wells were again washed, and a TMB substrate solution was added for color development in proportion to the bound phospho-FOXO3a or total FOXO3a present in the sample. The stop solution changed the color from blue to yellow, and the intensity of the color was measured at 450 nm.

### Slide preparation and fluorescent immunostaining of CHST15 and chondroitin 4-sulfate in lung melanomas

Mouse lung tissue dissected at the time of euthanasia was excised, photographed *en bloc* and divided into cassettes which were placed in 10% formalin in preparation for paraffin blocks and liquid nitrogen for further processing. Representative formalin-fixed tissues with melanomas from six control and six rhARSB-treated mice were sent to the Veterinary Diagnostic Laboratory at the University of Illinois Champagne-Urbana for sectioning and hematoxylin-eosin staining using standard procedures under the supervision of HW.

Immunostaining of chondroitin 4-sulfate and CHST15 in A375 cells was performed using the paraffin-embedded slides of six representative control and six representative ARSB-treated lung sections. Slides were heated on hot plate at 60 °C for 2h. The lung tissue sections on the slides were deparaffinized by immersing in Xylene 3x each for 5 min, 100% ethanol 2x each for 3 min, 95% ethanol 2x each for 3 min, and 70% ethanol 3 min. Antigen was retrieved using citrate-based antigen unmasking solution (H-3300–250, Vector Laboratories, Inc), diluting 100x of antigen unmasking solution in H_2_O and incubating for 30 min in a water bath at 65 °C and cooling for 1h at room temperature. Slides were washed with PBST x3 each for 3 min. The tissue area was enclosed using ImmEdge Hydrophobic Barrier PAP Pen (H-4000, Vector Laboratories). The slides were incubated with 3% H_2_O_2_ for 10 min at room temperature and washed with PBST 3 times (3 min each) followed by 0.5% Triton-100 incubation for 10 min at room temperature and washed with PBST 3 times (3 min each). Then, background was blocked with 10% goat serum or horse serum for 1h at room temperature. Primary antibodies were diluted 1:333 for C4S (LY111, A3143, TCI) with 3% goat serum in 1% BSA and 1:50 for CHST15 (ab 224399, Abcam) with 3% horse serum. Primary antibodies (150 μl) were applied to each section and incubated overnight in a moist slide chamber at 4 °C. Slides were washed with PBST 3 times (3 min each). Fluorescein isothiocyanate (FITC)-labelled secondary antibody (Abcam) was diluted 1:500 with 3% goat or horse serum in 1% BSA, and applied to each section and incubated for 1 h in a moist slide chamber at RT. Slides were washed with PBST 4 times (3 min each), then mounted with prolong antifade mountant with DAPI (Thermo Fisher) and viewed after 10 min in the EVOS M5000 Imaging System (Thermo Fisher) at 20x with optimized and consistent settings. The captured TIFF images were exported for analysis and reproduction.

### ELISAs for BCL2, cytosolic cytochrome C, and cleaved caspase-3 (Asp175)

BCL2 in representative control and treated melanoma tissues and in A375 and normal melanocyte cell extracts were detected, using a commercial sandwich Duoset ELISA (DYC827B, R&D Systems). To measure cytosolic cytochrome C, the cytosolic fraction was isolated from A375 cells using a mitochondria isolation kit (Thermo Fisher), and the cytosolic (non-mitochondrial fraction) cytochrome *C* level was assayed using a cytochrome *C* ELISA kit (Quantikine ELISA, R&D Systems). Cleaved Caspase-3 (Asp175) in the lysates was measured by a commercial sandwich DuoSet ELISA (DYC835, R&D Systems).

### Activated caspase-3/7 live cell imaging

A375 cells were seeded (10,000 cells/well in 24-well plates) in routine growth media for 24h. Then, the media was exchanged and replenished with fresh media with or without 1 ng/ml rhARSB and Caspase-3/7 dye (Cat #4440, Incucyte Caspase-3/7 Green Dye, Sartorius) at a final concentration of 5 μM. The Caspase 3/7 dye-treated cells were scanned using EVOS M5000 Imaging System (Thermo Fisher) at 20x after 2h, 12h, and 24h of exposure to the dye and 48 h after treatment with rhARSB. The captured TIFF images were exported for analysis and reproduction.

### Oligonucleotide-based ELISA to detect nuclear Sp1

Oligonucleotide-binding assay (#41296, TransAM kit, Active Motif) was used to detect nuclear Sp1 in the tumor tissue, per recommendations.

### Determination of nuclear SMAD3 (pS423/S425)

SMAD3 (pS423/S425) in the treated or control nuclear extracts was determined by a commercial SMAD3 (pS423/S425) ELISA Kit (ab186038, Abcam), per recommendations.

### QRT-PCR for COP1, BCL2, CHST15 and CHST11

Total RNA was prepared from treated and control cells using an RNeasy Mini Kit (Qiagen). Equal amounts of purified RNAs from the control and treated cells were reverse-transcribed and amplified using Brilliant SYBR Green QRT-PCR Master Mix (Bio-Rad). Human β-actin was used as an internal control. QRT-PCR was performed using the following specific primers:

Human CHST11 (NM_018413) forward: 5′-GTTGGCAGAAGAAGCAGAGG-3′ and reverse: 5′-GACATAGAGGAGGGCAAGGA-3′;

Human CHST15 (NM_015892) forward: 5′-ACTGAAGGGAACGAAAACTGG-3′ and reverse: 5′-CCGTAATGGAAAGGTGATGAG-3′;

Human COP1 (NM_022457) left 5′-GTCAGTGAGGATAGCACAGTGC and right 5′-GAGAACTGCCACTGAAACCTGG-3′;

Human BCL2 (NM_000633) left 5′-ATCGCCCTGTGGATGACTGAGT-3′ and right 5′-GCCAGGAGAAATCAAACAGAGGC-3′;

Human Wnt5A (NM_003392) left 5′- TACGAGAGTGCTCGCATCCTCA-3′ and right 5′-TGGTCTTCAGGCTACATGAGCCG-3′;

Mouse CHST11 (NM_021439) left: 5′-TCAATGCCCAAACCTATTCC-3′ and right: 5′-CAAAACCCCACAAAAACACA-3′;

Mouse CHST15 (NM_029935.6) left: 5′-TATGCCCAGCGTAGAGAAGG-3′ and right: 5′- ACCAGCCAGAACCAAAAACA-3′;

Mouse COP1 (NM_011931) left: 5′-GCCTCTACTCTCTCAGTGA -3′ and right: 5′- GTCCCACTGAAACCTGGAGGTT-3′;

Mouse BCL2 (NM_009741) left: 5′-CCTGTGGATTGAGTACCTG-3′ and right 5′-AGCCAGGAGAAATCAAACAGAGG-3′;

Human β-actin (NM_001101) left: 5′-CACCATTGGCAATGAGCGGTTC-3′ and right: 5′-AGGTCDTTTGCGGATGTCCACGT-3′;

Mouse β-actin (NM_007393) left: 5′-CATTGCTGACAGGATGCAGAAGG-3′ and right: 5′-TGCTGGAAGGTGGACAGTGAGG-3′.

Cycle threshold (Ct) was determined during the exponential phase of amplification, as described previously (30). Fold changes in expression were determined by the difference between the Ct values of the gene of interest and the control.

### Transcription Factor filter plate assay for c-Myc and ETS1

Transcription Factor (TF) Filter Plate Assay kit and specific labeled DNA probes for c-Myc and for ETS1 were obtained (Signosis). Nuclear extracts from treated and control cells were mixed with a specific biotin-labeled ETS1 or c-Myc DNA binding sequence and allowed to form TF-DNA complexes. A filter plate retained the bound DNA probe and removed the free probe. The bound, pre-labeled DNA probe was then eluted from the filter and collected for quantitative analysis by DNA plate hybridization and detected with streptavidin-HRP. Luminescence was reported as relative light units (RLUs) on a microplate reader. The luminescence was normalized by total protein of the nuclear extract and expressed as percentage of control.

### BCL2 and CHST11 promoter activity

BCL2 and CHST11 promoter activity of A375 melanoma cells following rhARSB or ARSB siRNA was determined using human BCL2 and CHST11 promoter constructs in a *Renilla reniformis* luciferase reporter gene (Ren SP, Origene). Transfections were performed with cells at 70% confluence, using FuGENE HD transfection reagent (Promega). The β-actin promoter (GoClone) construct was used as positive control and a scrambled sequence (R01) with the *Renilla* luciferase reporter was a negative control. After incubation for 24h, luminescence was read in a microplate reader (BMG). The changes in luminescence due to modification of binding to the BCL2 or the CHST11 promoter were detected by the LightSwitch assay system (SwitchGear Genomics).

### Chromatin immunoprecipitation (ChIP) assay of FOXO3a with COP1 promoter sequence

ChIP assay was performed utilizing a ChIP assay kit (#53008, Active Motif). Control and treated A375 melanoma cells were fixed with 1% formaldehyde for 10 min at room temperature. This was followed by the shearing of chromatin by sonication on ice to obtain DNA lengths between 200 and 1000 base pairs. Soluble chromatin fragments of 200 to 1000 bp in length were incubated with 2 μg of FOXO3a polyclonal antibody (#720128, Invitrogen, Thermo Fisher) at 4 °C overnight. Rabbit IgG was used as a negative control for validating the ChIP assay. Protein–DNA complexes were precipitated by protein G-coupled magnetic beads. DNA was purified from the immunoprecipitated complexes by a reversal of cross-linking and followed by proteinase K treatment. Real-time RT-PCR was performed using SYBR Green QRT-PCR master mix (Bio-Rad) with the primer pair (left: 5′-AGTGCCTTCTACTCCGCTTT-3′ and right: 5′- GACATCGTGACTCCCTCCC -3′) which encompassed the putative FOXO3a binding site in the COP1 promoter. The ChIP qPCR result was calculated using the ΔΔCt method. The Ct value of each ChIP fraction was normalized to the input DNA fraction and expressed as % Input. The PCR-end products of different treatment groups were run on a 2% Agarose gel for the analysis of amplified product density and size.

### Western blots of phospho(Ser473)-AKT1 and pan-AKT

Cell lysates were prepared from control and treated cells in cell lysis buffer (Cell Signaling) with protease and phosphatase inhibitors (Halt Protease and Phosphatase Inhibitor Cocktail, Thermo Fisher Scientific). Western blots were performed on 12% SDS gels with commercial antibodies to phospho-(Ser473)-AKT protein (1:1000; Cell Signaling, #4060), AKT (pan) (1:1000; Cell Signaling, #4691), α-tubulin (1:1000; Cell Signaling, #2125) and GAPDH (1:1000; Cell Signaling, #97166) to probe for the proteins of interest by established procedures (30). Immunoreactive bands were localized by anti-rabbit or anti-mouse–HRP conjugate (1:2000; #w4018, #w4021, Promega Corporation) and visualized using enhanced chemiluminescence (Clarity Western ECL Substrate; Bio-Rad) in Odyssey XFImaging System (LI-COR). Optical density of immunoreactive bands was measured, and the optical densities of phospho-(Ser473)-AKT protein bands were normalized with the optical densities AKT (pan) protein and α-tubulin or GAPDH control. The density of treated and control samples was then compared.

### Sulfated glycosaminoglycan and chondroitin 4-sulfate assays

Total sulfated glycosaminoglycan (GAG) content in cell lysates was measured using the sulfated GAG assay (Blyscan, Biocolor Ltd), as previously described (30). This assay detects heparin, heparan sulfate, chondroitin sulfates, dermatan sulfate, and keratan sulfate, but not disaccharides or unsulfated GAGs.

### Disaccharide analysis

Disaccharide analysis was performed as detailed previously (32). Unsaturated disaccharide standards of CS (ΔUA-GalNAc; ΔUA-GalNAc4S; ΔUA-GalNAc6S; ΔUA2S-GalNAc; ΔUA2S-Gal-NAc4S; ΔUA2S-GalNAc6S; ΔUA-GalNAc4S6S; ΔUA2S-GalNAc4S6S), unsaturated disaccharide standards of HS (ΔUA-GlcNAc; ΔUA-GlcNS; ΔUA-GlcNAc6S; ΔUA2S-GlcNAc; ΔUA2S-GlcNS; ΔUA-GlcNS6S; ΔUA2S-GlcNAc6S; ΔUA2S-GlcNS6S), and unsaturated disaccharide standard of HA (ΔUA-GlcNAc), where ΔUA is 4-deoxy-α-L-threo-hex-4-enopyranosyluronic acid, were purchased from Iduron. Actinase E was obtained from Kaken Biochemicals (Japan). Chondroitin lyase ABC from *Proteus vulgaris* was expressed in laboratory. Recombinant *Flavobacterial* heparin lyases I, II, and III were expressed in our laboratory (JY, FZ) using *Escherichia coli* strains provided by Jian Liu (College of Pharmacy, University of North Carolina). 2-Aminoacridone (AMAC) and sodium cyanoborohydride were obtained from Sigma-Aldrich. All solvents were HPLC grade.

### GAG preparation for disaccharide analysis of A375 cells, AMAC labeling of A375-derived samples, and LC-MS of A375-derived samples

All the samples were proteolyzed at 55 °C with 200 μl of 5 mg/ml actinase E for 24 h and followed by actinase E deactivation at 100 °C for 30 min and processed as previously (32). The dried samples were AMAC-labeled by adding 10 μl of 0.1 M AMAC in DMSO/acetic acid (17/3, v/v) incubating at room temperature for 10 min, followed by adding 10 μl of 1 M aqueous sodium cyanoborohydride and incubating for 1 h at 45 °C. A mixture containing all 17-disaccharide standards prepared at 1 ng/μl was similarly AMAC-labeled and used for each run as an external standard. After the AMAC-labeling reaction, the samples were centrifuged, and each supernatant was recovered. Liquid chromatography (LC) was performed on an Agilent 1200 LC system at 45 °C using an Agilent Poroshell 120 ECC18 (2.7 μm, 3.0 × 50 mm) column, as previously.

Data are presented as the overall GAG content of the 8 cell samples (A375 control, ARSB si, rhARSB, and control si each in duplicate). The % of each detected CS disaccharide is presented for each cell sample. Concentrations in ng/μl was determined by knowing the concentration of each disaccharide standard in every MRM analysis. From the mass spectrometry results, we obtained the peak area for each disaccharide component in both the standards and the samples. We then calculated the concentration of each disaccharide in the samples using the formula: concentration of sample = (peak area of sample/peak area of standard) × concentration of standard. The chondroitin disaccharide concentrations were also expressed as ng/ml of sample and as ng/mg of protein, using protein concentrations in mg/ml of replicate samples.

### Statistical analysis

Data presented are the mean ± SD of at least six independent experiments, except if stated otherwise. Statistical significance of two-way comparisons was determined by unpaired, two-tailed *t*-tests with unequal standard deviations using Excel or Prizm (Version 10.3, GraphPad) software, unless stated otherwise. In the figures, specific *p*-values are indicated.

## Data availability

The data generated in this study are available upon request from the corresponding author.

## Supporting information

This article contains supporting information.

## Conflict of interest

The authors declare that they have no conflicts of interest with the contents of this article.

## Supporting information

Supporting Informations

## References

[1] Arnold M., Singh D., Laversanne M., Vignat J., Vaccarella S., Meheus F. (2022). Global burden of cutaneous melanoma in 2020 and projections to 2040. JAMA. Dermatol..

[2] Sung H., Ferlay J., Siegel R.L., Laversanne M., Soerjomataram I., Jemal A. (2021). Global cancer statistics 2020: GLOBOCAN estimates of incidence and mortality worldwide for 36 cancers in 185 countries. CA. Cancer. J. Clin..

[3] Arnold M., de Vries E., Whiteman D.C., Jemal A., Bray F., Parkin D.M. (2018). Global burden of cutaneous melanoma attributable to ultraviolet radiation in 2012. Int. J. Cancer..

[4] Al-Sadek T., Yusuf N. (2024). Ultraviolet radiation: biological and medical implications. Curr. Issues. Mol. Biol..

[5] Zhang Q., Lin L., Fang F., Cui B., Zhu C., Luo S. (2023). Dissecting the functions of COP1 in the UVR8 pathway with a COP1 variant in Arabidopsis. Plant. J..

[6] Yadav A., Singh D., Lingwan M., Yadukrishnan P., Masakapalli S.K., Datta S. (2020). Light signaling and UV-B-mediated plant growth regulation. J. Integr. Plant. Biol..

[7] Kinyó A., Kiss-László Z., Hambalkó S., Bebes A., Kiss M., Széll M. (2010). COP1 contributes to UVB-induced signaling in human keratinocytes. J. Invest Dermatol..

[8] Fazekas B., Polyánka H., Bebes A., Tax G., Szabó K., Farkas K. (2014). UVB-dependent changes in the expression of fast-responding early genes is modulated by huCOP1 in keratinocytes. J. Photochem. Photobiol. B.

[9] Favory J.J., Stec A., Gruber H., Rizzini L., Oravecz A., Funk M. (2009). Interaction of COP1 and UVR8 regulates UV-B-induced photomorphogenesis and stress acclimation in Arabidopsis. EMBO J..

[10] Wang Y., Wang L., Guan Z., Chang H., Ma L., Shen C. (2022). Structural insight into UV-B-activated UVR8 bound to COP1. Sci. Adv..

[11] Mankotia S., Jakhar P., Satbhai S.B. (2024). HY5: a key regulator for light-mediated nutrient uptake and utilization by plants. New. Phytol..

[12] Lau K., Podolec R., Chappuis R., Ulm R., Hothorn M. (2019). Plant photoreceptors and their signaling components compete for COP1 binding via VP peptide motifs. EMBO J..

[13] Dornan D., Wertz I., Shimizu H., Arnott D., Frantz G.D., Dowd P. (2004). The ubiquitin ligase COP1 is a critical negative regulator of p53. Nature.

[14] Bianchi E., Denti S., Catena R., Rossetti G., Polo S., Gasparian S. (2003). Characterization of human constitutive photomorphogenesis protein 1, a RING finger ubiquitin ligase that interacts with Jun transcription factors and modulates their transcriptional activity. J. Biol. Chem..

[15] Xie Y., Cao Z., Wong E.W., Guan Y., Ma W., Zhang J.Q. (2018). COP1/DET1/ETS axis regulates ERK transcriptome and sensitivity to MAPK inhibitors. J. Clin. Invest..

[16] Marine J.C. (2012). Spotlight on the role of COP1 in tumorigenesis. Nat. Rev. Cancer..

[17] Song Y., Liu Y., Pan S., Xie S., Wang Z.W., Zhu X. (2020). Role of the COP1 protein in cancer development and therapy. Semin. Cancer. Biol..

[18] Vitari A.C., Leong K.G., Newton K., Yee C., O'Rourke K., Liu J. (2011). COP1 is a tumour suppressor that causes degradation of ETS transcription factors. Nature.

[19] Ducker C., Shaw P.E. (2021). Ubiquitin-mediated control of ETS transcription factors: roles in cancer and development. Int. J. Mol. Sci..

[20] Yu Z., Shah D.M. (2007). Curcumin down-regulates ETS1 and Bcl-2 expression in human endometrial carcinoma HEC-1-A cells. Gynecol. Oncol..

[21] Huang L., Zhai Y., La J., Lui J.W., Moore S.P.G., Little E.C. (2021). Targeting pan-ETS factors inhibits melanoma progression. Cancer. Res..

[22] Dong L., Jiang C.C., Thorne R.F., Croft A., Yang F., Liu H. (2011). ETS1 mediates upregulation of Mcl-1 downstream of XBP-1 in human melanoma cells upon ER stress. Oncogene.

[23] Bhattacharyya S., O-Sullivan I., Tobacman J.K. (2024). N-Acetylgalactosamine-4-sulfatase (Arylsulfatase B) regulates PD-L1 expression in melanoma by an HDAC3-mediated epigenetic mechanism. Int. J. Mol. Sci..

[24] Bhattacharyya S., Feferman L., Terai K., Dudek A.Z., Tobacman J.K. (2017). Decline in arylsulfatase B leads to increased invasiveness of melanoma cells. Oncotarget.

[25] Bhattacharyya S., O-Sullivan I., Tu J., Chen Z., Tobacman J.K. (2024). Exogenous recombinant N-acetylgalactosamine-4-sulfatase (Arylsulfatase B; ARSB) inhibits progression of B16F10 cutaneous melanomas and modulates cell signaling. Biochim. Biophys. Acta. Mol. Basis. Dis..

[26] Tobacman J.K., Bhattacharyya S. (2022). Profound impact of decline in N-acetyflgalactosamine-4-sulfatase (arylsulfatase B) on molecular pathophysiology and human diseases. Int. J. Mol. Sci..

[27] Bhattacharyya S., Tobacman J.K. (2012). Hypoxia reduces arylsulfatase B activity and silencing arylsulfatase B replicates and mediates the effects of hypoxia. PLoS One.

[28] Bhattacharyya S., Feferman L., Tobacman J.K. (2016). Inhibition of phosphatase activity follows decline in sulfatase activity and leads to transcriptional effects through sustained phosphorylation of transcription factor MITF. PLoS One.

[29] Bhattacharyya S., Feferman L., Tobacman J.K. (2019). Dihydrotestosterone inhibits arylsulfatase B and Dickkopf Wnt signaling pathway inhibitor (DKK)-3 leading to enhanced Wnt signaling in prostate epithelium in response to stromal Wnt3A. Prostate.

[30] Bhattacharyya S., Feferman L., Tobacman J.K. (2014). Arylsulfatase B regulates versican expression by galectin-3 and AP-1 mediated transcriptional effects. Oncogene.

[31] Bhattacharyya S., Feferman L., Tobacman J.K. (2014). Increased expression of colonic Wnt9A through Sp1-mediated transcriptional effects involving arylsulfatase B, chondroitin 4-sulfate, and galectin-3. J. Biol. Chem..

[32] Bhattacharyya S., Feferman L., Han X., Xia K., Zhang F., Linhardt R.J. (2020). Increased CHST15 follows decline in arylsulfatase B (ARSB) and disinhibition of non-canonical WNT signaling: potential impact on epithelial and mesenchymal identity. Oncotarget.

[33] Ye J., Suizu F., Yamakawa K., Mukai Y., Yoneyama H., Kondo J. (2024). Intra-tumoral administration of CHST15 siRNA remodels tumor microenvironment and augments tumor-infiltrating T cells in pancreatic cancer. Mol. Ther. Oncol..

[34] Chen Y., Zhang Y., Wang Z., Wang Y., Luo Y., Sun N. (2022). CHST15 gene germline mutation is associated with the development of familial myeloproliferative neoplasms and higher transformation risk. Cell. Death. Dis..

[35] Ito Z., Takakura K., Suka M., Kanai T., Saito R., Fujioka S. (2017). Prognostic impact of carbohydrate sulfotransferase 15 in patients with pancreatic ductal adenocarcinoma. Oncol. Lett..

[36] ten Dam G.B., van de Westerlo E.M., Purushothaman A., Stan R.V., Bulten J., Sweep F.C. (2007). Antibody GD3G7 selected against embryonic glycosaminoglycans defines chondroitin sulfate-E domains highly up-regulated in ovarian cancer and involved in vascular endothelial growth factor binding. Am. J. Pathol..

[37] Habuchi O. (2022). Functions of chondroitin/dermatan sulfate containing GalNAc4,6-disulfate. Glycobiology.

[38] Nadanaka S., Tamura J.-I., Kitagawa H. (2022). Chondroitin sulfates control invasiveness of the basal-like breast cancer cell line MDA-MB-231 through ROR1. Front. Oncol..

[39] Brunet A., Bonni A., Zigmond M.J., Lin M.Z., Juo P., Hu L.S. (1999). Akt promotes cell survival by phosphorylating and inhibiting a forkhead transcription factor. Cell.

[40] Dan Y., Chen L., Jin S., Xing X., Zhu Y., Jiang M. (2024). Photobiomodulation using 830 nm lighting-emitting diode inhibits melanogenesis via FOXO3a in human melanocyte. Pigment. Cell. Melanoma. Res..

[41] Liu Y., Ao X., Ding W., Ponnusamy M., Wu W., Hao X. (2018). Critical role of FOXO3a in carcinogenesis. Mol. Cancer..

[42] Ma C., Xie L. (2024). Prognostic model development and clinical correlation of eight key genes in skin cutaneous melanoma. Heliyon.

[43] Yan F., Liao R., Silva M., Li S., Jiang Y., Peng T. (2020). Pristimerin-induced uveal melanoma cell death via inhibiting PI3K/Akt/FoxO3a signalling pathway. J. Cell. Mol. Med..

[44] Kim J.H., Oh C.T., Kwon T.R., Kim J.H., Bak D.H., Kim H. (2020). Inhibition of melanogenesis by sodium 2-mercaptoethanesulfonate. Korean. J. Physiol. Pharmacol..

[45] Bai M., Zhang M., Long F., Yu N., Zeng A., Zhao R. (2017). Circulating microRNA-194 regulates human melanoma cells via PI3K/AKT/FoxO3a and p53/p21 signaling pathway. Oncol. Rep..

[46] Kim J., Choi H., Cho E.G., Lee T.R. (2014). FoxO3a is an antimelanogenic factor that mediates antioxidant-induced depigmentation. J. Invest. Dermatol..

[47] Hilmi C., Larribere L., Deckert M., Rocchi S., Giuliano S., Bille K. (2008). Involvement of FKHRL1 in melanoma cell survival and death. Pigment. Cell. Melanoma. Res..

[48] Kipps T.J. (2022). ROR1: an orphan becomes apparent. Blood.

[49] Quezada M.J., Lopez-Bergami P. (2023). The signaling pathways activated by ROR1 in cancer. Cell. Signal..

[50] Fernández N.B., Lorenzo D., Picco M.E., Barbero G., Dergan-Dylon L.S., Marks M.P. (2016). ROR1 contributes to melanoma cell growth and migration by regulating N-cadherin expression via the PI3K/Akt pathway. Mol. Carcinog..

[51] Mao Y., Xu L., Wang J., Zhang L., Hou N., Xu J. (2019). ROR1 associates unfavorable prognosis and promotes lymphoma growth in DLBCL by affecting PI3K/Akt/mTOR signaling pathway. Biofactors.

[52] Zhang S., Chen L., Cui B., Chuang H.Y., Yu J., Wang-Rodriguez J. (2012). ROR1 is expressed in human breast cancer and associated with enhanced tumor-cell growth. PLoS One.

[53] Choi M.Y., Widhopf G.F., Wu C.C., Cui B., Lao F., Sadarangani A. (2015). Pre-clinical specificity and safety of UC-961, a first-in-class monoclonal antibody targeting ROR1. Clin. Lymphoma. Myeloma. Leuk..

[54] Shatsky R.A., Batra-Sharma H., Helsten T., Schwab R.B., Pittman E.I., Pu M. (2024). A phase 1b study of zilovertamab in combination with paclitaxel for locally advanced/unresectable or metastatic Her2-negative breast cancer. Breast. Cancer. Res..

[55] Liu D., Kaufmann G.F., Breitmeyer J.B., Dickson K.A., Marsh D.J., Ford C.E. (2022). The anti-ROR1 monoclonal antibody Zilovertamab inhibits the proliferation of ovarian and endometrial cancer cells. Pharmaceutics.

[56] Li L., Huang W., Ren X., Wang Z., Ding K., Zhao L. (2024). Unlocking the potential: advancements and future horizons in ROR1-targeted cancer therapies. Sci. China. Life. Sci..

[57] Hasan M.K., Widhopf G.F., Zhang S., Lam S.M., Shen Z., Briggs S.P. (2019). Wnt5a induces ROR1 to recruit cortactin to promote breast-cancer migration and metastasis. NPJ Breast. Cancer..

[58] Byers S., Crawley A.C., Brumfield L.K., Nuttall J.D., Hopwood J.J. (2000). Enzyme replacement therapy in a feline model of MPS VI: modification of enzyme structure and dose frequency. Pediatr. Res..

[59] Bohnsack R.N., Misra S.K., Liu J., Ishihara-Aoki M., Pereckas M., Aoki K. (2024). Lysosomal enzyme binding to the cation-independent mannose 6-phosphate receptor is regulated allosterically by insulin-like growth factor 2. Sci. Rep..

[60] Belharazem D., Magdeburg J., Berton A.K., Beissbarth L., Sauer C., Sticht C. (2016). Carcinoma of the colon and rectum with deregulation of insulin-like growth factor 2 signaling: clinical and molecular implications. J. Gastroenterol..

[61] Lee E., Song C.H., Bae S.J., Ha K.T., Karki R. (2023). Regulated cell death pathways and their roles in homeostasis, infection, inflammation, and tumorigenesis. Exp. Mol. Med..

[62] Bedoui S., Herold M.J., Strasser A. (2020). Emerging connectivity of programmed cell death pathways and its physiological implications. Nat. Rev. Mol. Cell. Biol..

[63] Isoherranen K., Sauroja I., Jansen C., Punnonen K. (1999). UV irradiation induces downregulation of bcl-2 expression in vitro and in vivo. Arch. Dermatol. Res..

[64] Knezevic D., Zhang W., Rochette P.J., Brash D.E. (2007). Bcl-2 is the target of a UV-inducible apoptosis switch and a node for UV signaling. Proc. Natl. Acad. Sci. U. S. A..

[65] Mao P., Brown A.J., Esaki S., Lockwood S., Poon G.M.K., Smerdon M.J. (2018). ETS transcription factors induce a unique UV damage signature that drives recurrent mutagenesis in melanoma. Nat. Commun..

[66] Carrasco Pro S., Hook H., Bray D., Berenzy D., Moyer D., Yin M. (2023). Widespread perturbation of ETS factor binding sites in cancer. Nat. Commun..

