# Supplementary material for: Arylsulfatase B induces melanoma apoptosis by the ubiquitin ligase COP1
Source: J Biol Chem. 2025 Jun 23;301(8):110402. doi: 10.1016/j.jbc.2025.110402 (PMC12301740; doi:10.1016/j.jbc.2025.110402)
Supplement: Supporting Informations [file mmc1.pptx]

## Slide 1
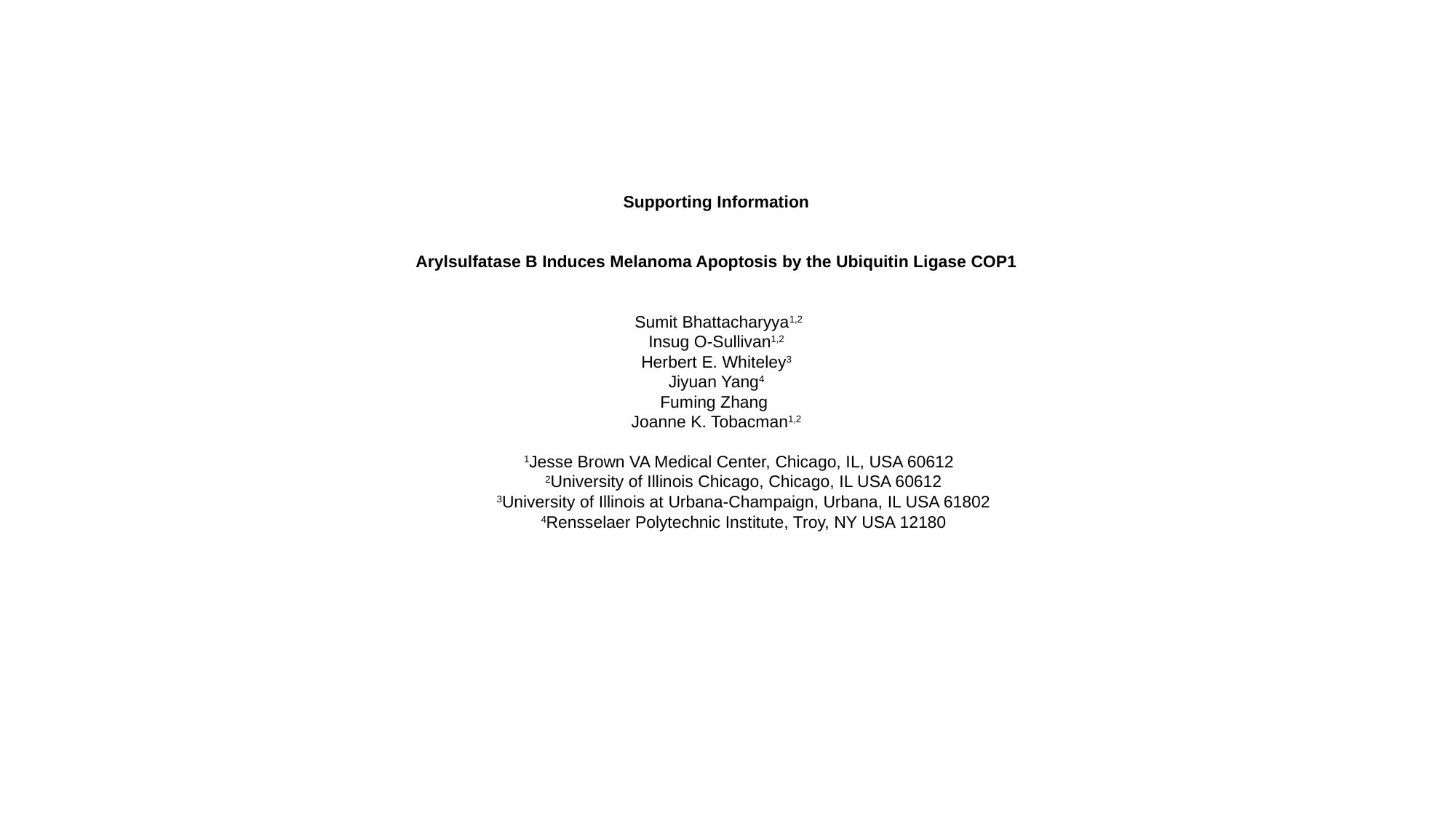

Supporting Information
Arylsulfatase B Induces Melanoma Apoptosis by the Ubiquitin Ligase COP1
 Sumit Bhattacharyya1,2
Insug O-Sullivan1,2
Herbert E. Whiteley3
Jiyuan Yang4
Fuming Zhang
Joanne K. Tobacman1,2
1Jesse Brown VA Medical Center, Chicago, IL, USA 60612
2University of Illinois Chicago, Chicago, IL USA 60612
3University of Illinois at Urbana-Champaign, Urbana, IL USA 61802
4Rensselaer Polytechnic Institute, Troy, NY USA 12180

## Slide 2
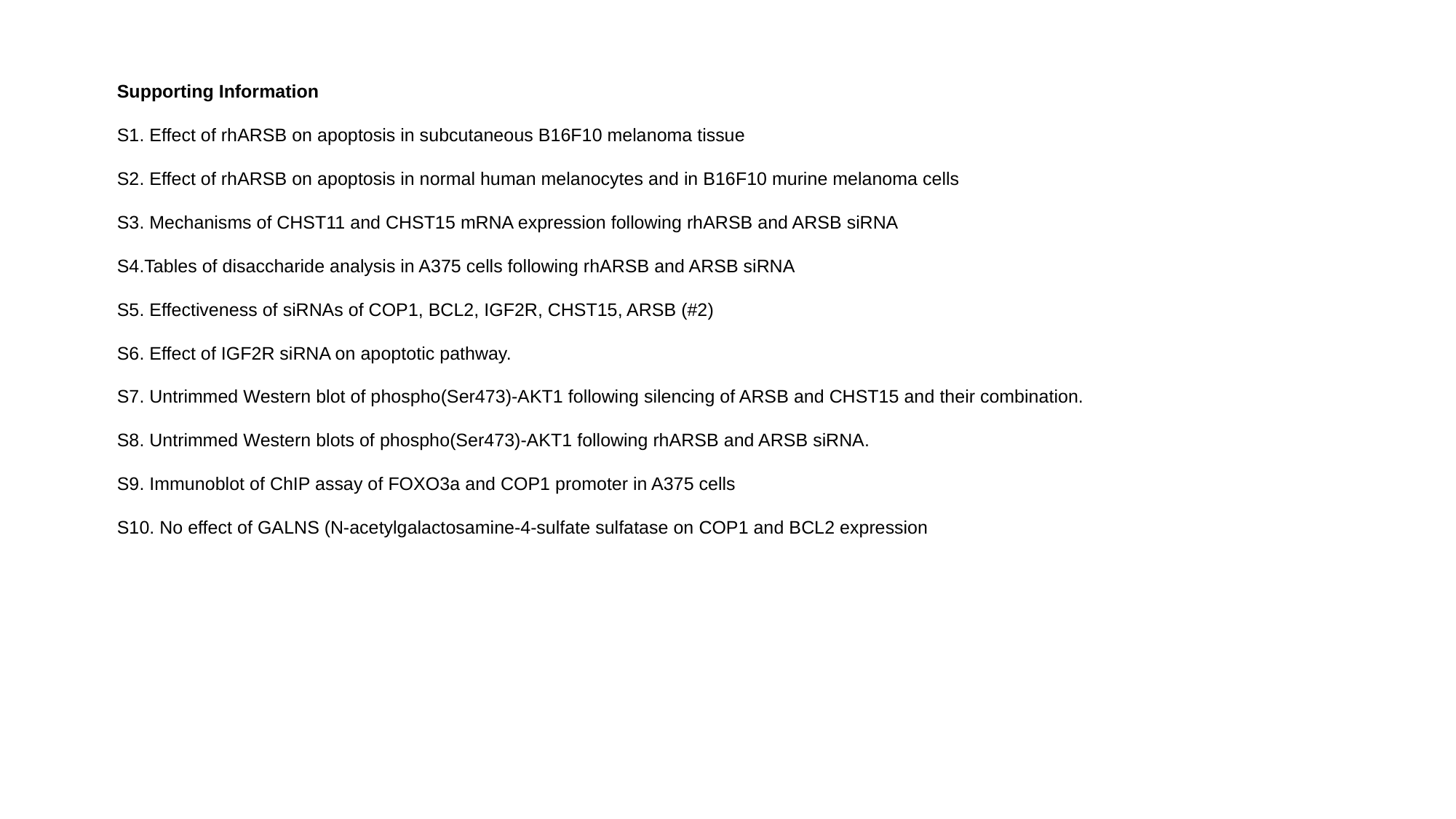

Supporting Information
S1. Effect of rhARSB on apoptosis in subcutaneous B16F10 melanoma tissue
S2. Effect of rhARSB on apoptosis in normal human melanocytes and in B16F10 murine melanoma cells
S3. Mechanisms of CHST11 and CHST15 mRNA expression following rhARSB and ARSB siRNA
S4.Tables of disaccharide analysis in A375 cells following rhARSB and ARSB siRNA
S5. Effectiveness of siRNAs of COP1, BCL2, IGF2R, CHST15, ARSB (#2)
S6. Effect of IGF2R siRNA on apoptotic pathway.
S7. Untrimmed Western blot of phospho(Ser473)-AKT1 following silencing of ARSB and CHST15 and their combination.
S8. Untrimmed Western blots of phospho(Ser473)-AKT1 following rhARSB and ARSB siRNA.
S9. Immunoblot of ChIP assay of FOXO3a and COP1 promoter in A375 cells
S10. No effect of GALNS (N-acetylgalactosamine-4-sulfate sulfatase on COP1 and BCL2 expression

## Slide 3
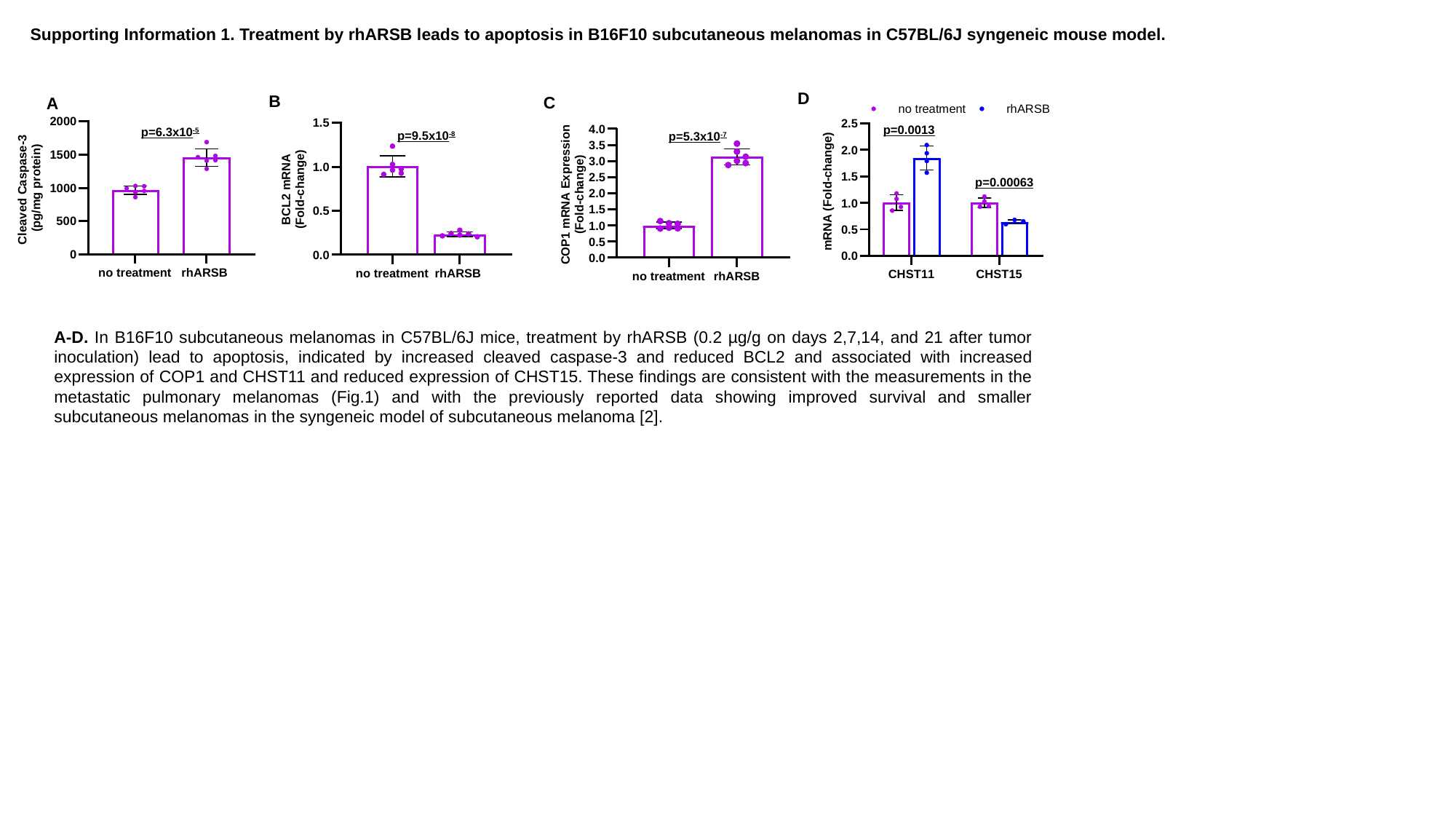

Supporting Information 1. Treatment by rhARSB leads to apoptosis in B16F10 subcutaneous melanomas in C57BL/6J syngeneic mouse model.
p=0.0013
p=0.00063
p=6.3x10^-5^
p=9.5x10^-8^
p=5.3x10^-7^
A-D. In B16F10 subcutaneous melanomas in C57BL/6J mice, treatment by rhARSB (0.2 µg/g on days 2,7,14, and 21 after tumor inoculation) lead to apoptosis, indicated by increased cleaved caspase-3 and reduced BCL2 and associated with increased expression of COP1 and CHST11 and reduced expression of CHST15. These findings are consistent with the measurements in the metastatic pulmonary melanomas (Fig.1) and with the previously reported data showing improved survival and smaller subcutaneous melanomas in the syngeneic model of subcutaneous melanoma [2].
D
B
C
A
Treatment by ARSB leads to apoptosis, mediated by COP1 and CHST15.

## Slide 4
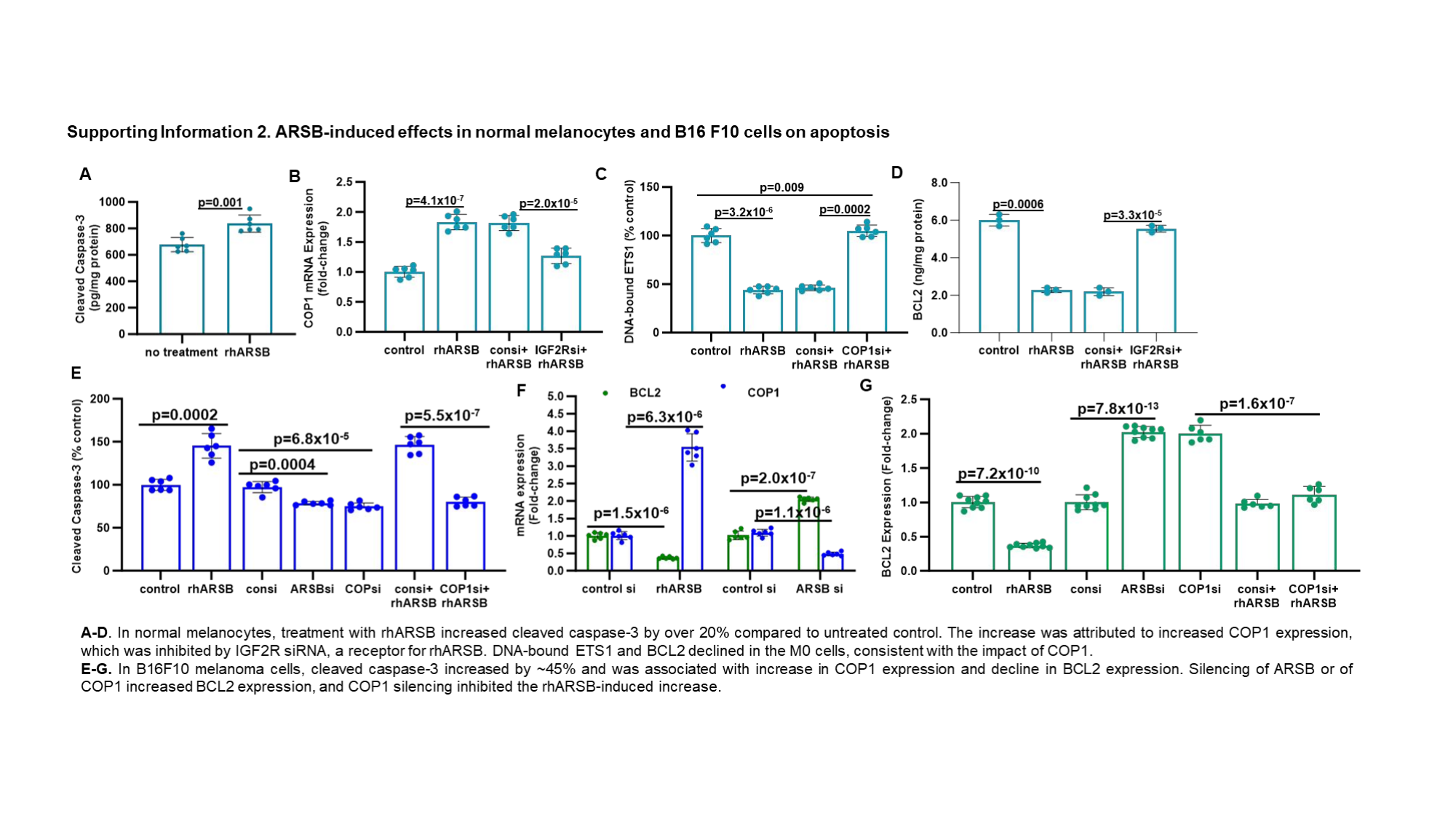

## Slide 5
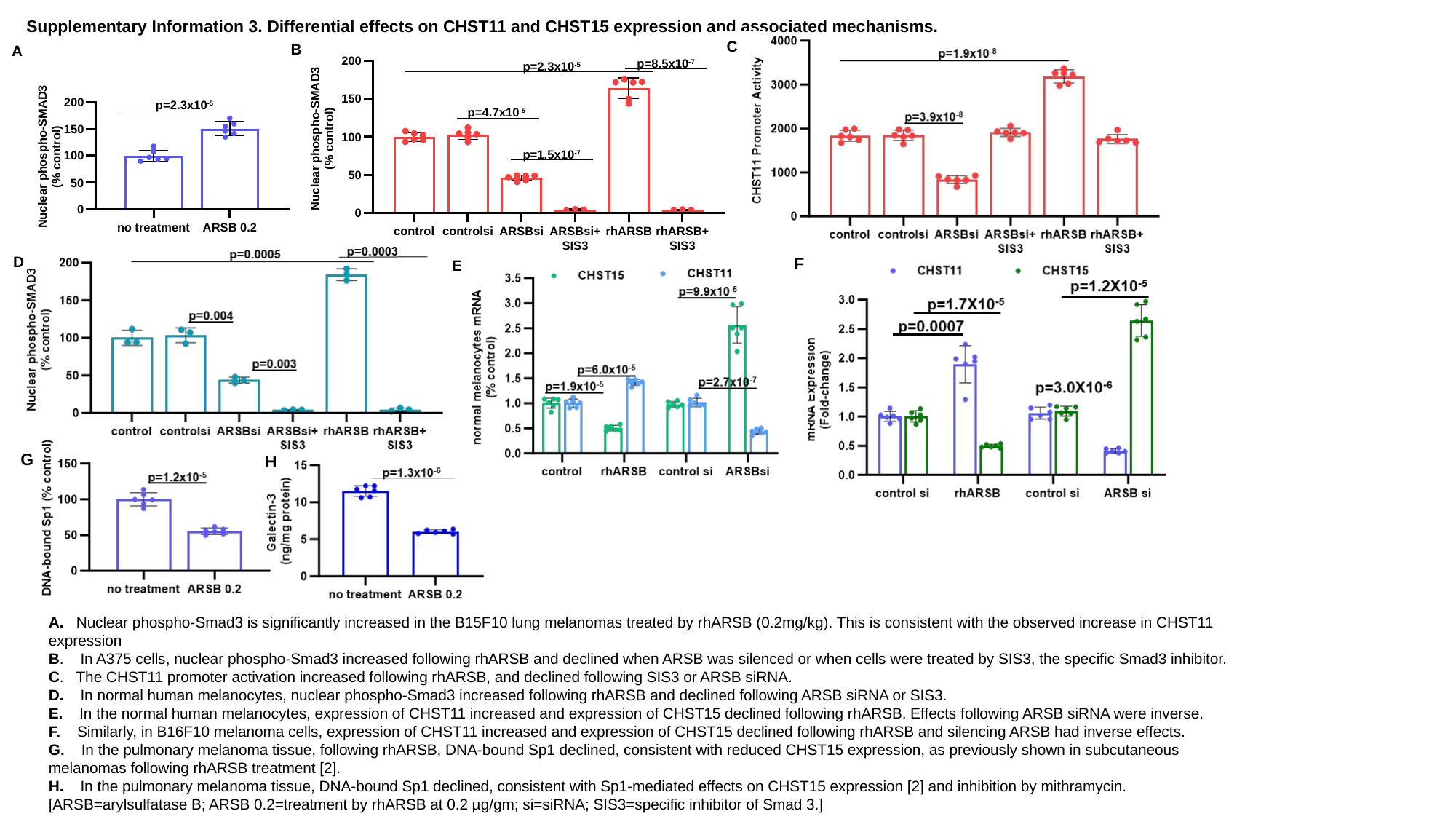

Supplementary Information 3. Differential effects on CHST11 and CHST15 expression and associated mechanisms.
C.
A.
p=2.3x10^-5^
p=8.5x10^-7^
p=2.3x10^-5^
p=4.7x10^-5^
p=1.5x10^-7^
F
C
B
B.
A
D
E
A. Nuclear phospho-Smad3 is significantly increased in the B15F10 lung melanomas treated by rhARSB (0.2mg/kg). This is consistent with the observed increase in CHST11 expression
B. In A375 cells, nuclear phospho-Smad3 increased following rhARSB and declined when ARSB was silenced or when cells were treated by SIS3, the specific Smad3 inhibitor.
C. The CHST11 promoter activation increased following rhARSB, and declined following SIS3 or ARSB siRNA.
D. In normal human melanocytes, nuclear phospho-Smad3 increased following rhARSB and declined following ARSB siRNA or SIS3.
E. In the normal human melanocytes, expression of CHST11 increased and expression of CHST15 declined following rhARSB. Effects following ARSB siRNA were inverse.
F. Similarly, in B16F10 melanoma cells, expression of CHST11 increased and expression of CHST15 declined following rhARSB and silencing ARSB had inverse effects.
G. In the pulmonary melanoma tissue, following rhARSB, DNA-bound Sp1 declined, consistent with reduced CHST15 expression, as previously shown in subcutaneous melanomas following rhARSB treatment [2].
H. In the pulmonary melanoma tissue, DNA-bound Sp1 declined, consistent with Sp1-mediated effects on CHST15 expression [2] and inhibition by mithramycin.
[ARSB=arylsulfatase B; ARSB 0.2=treatment by rhARSB at 0.2 µg/gm; si=siRNA; SIS3=specific inhibitor of Smad 3.]
G
H

## Slide 6
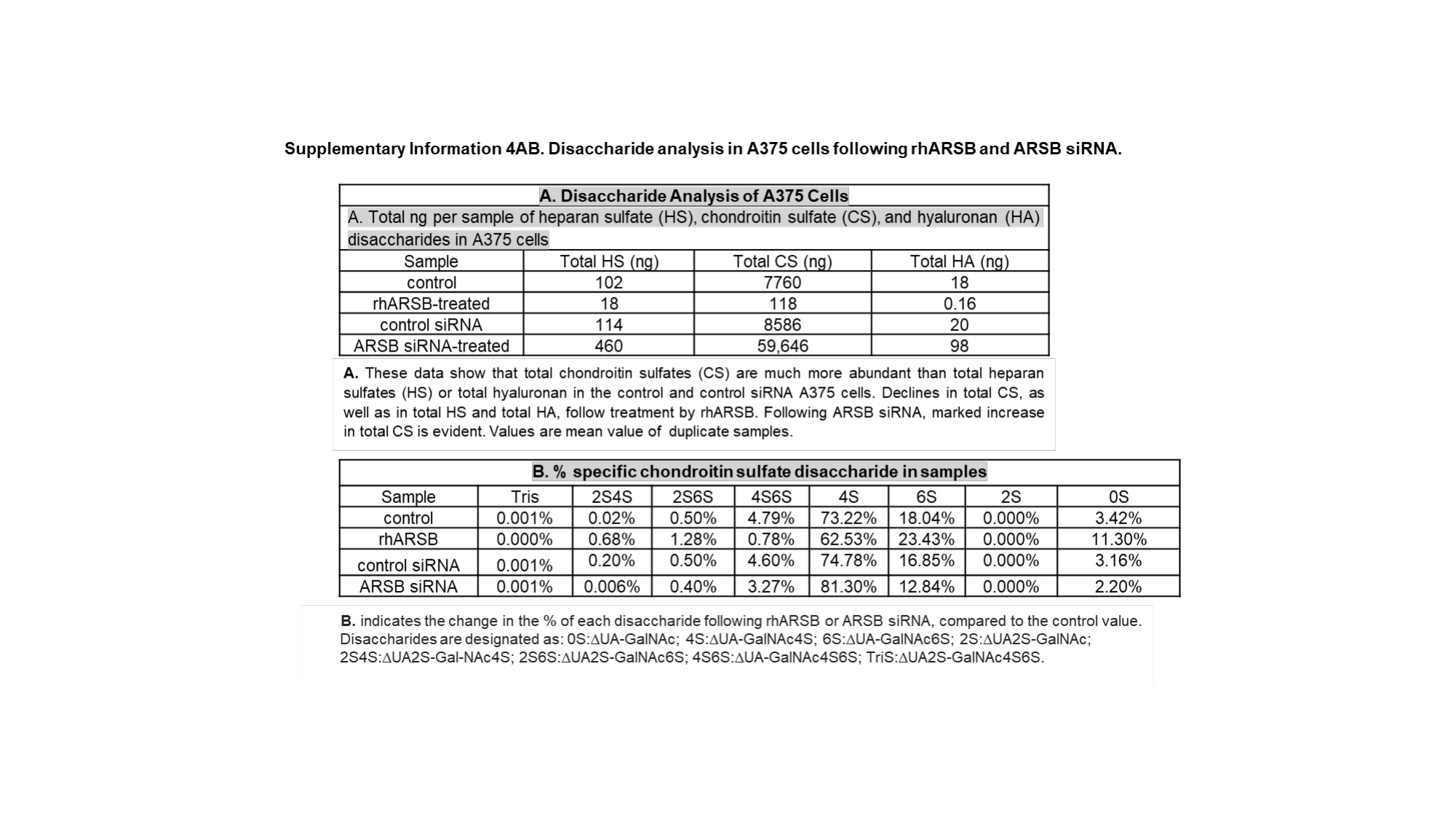

## Slide 7
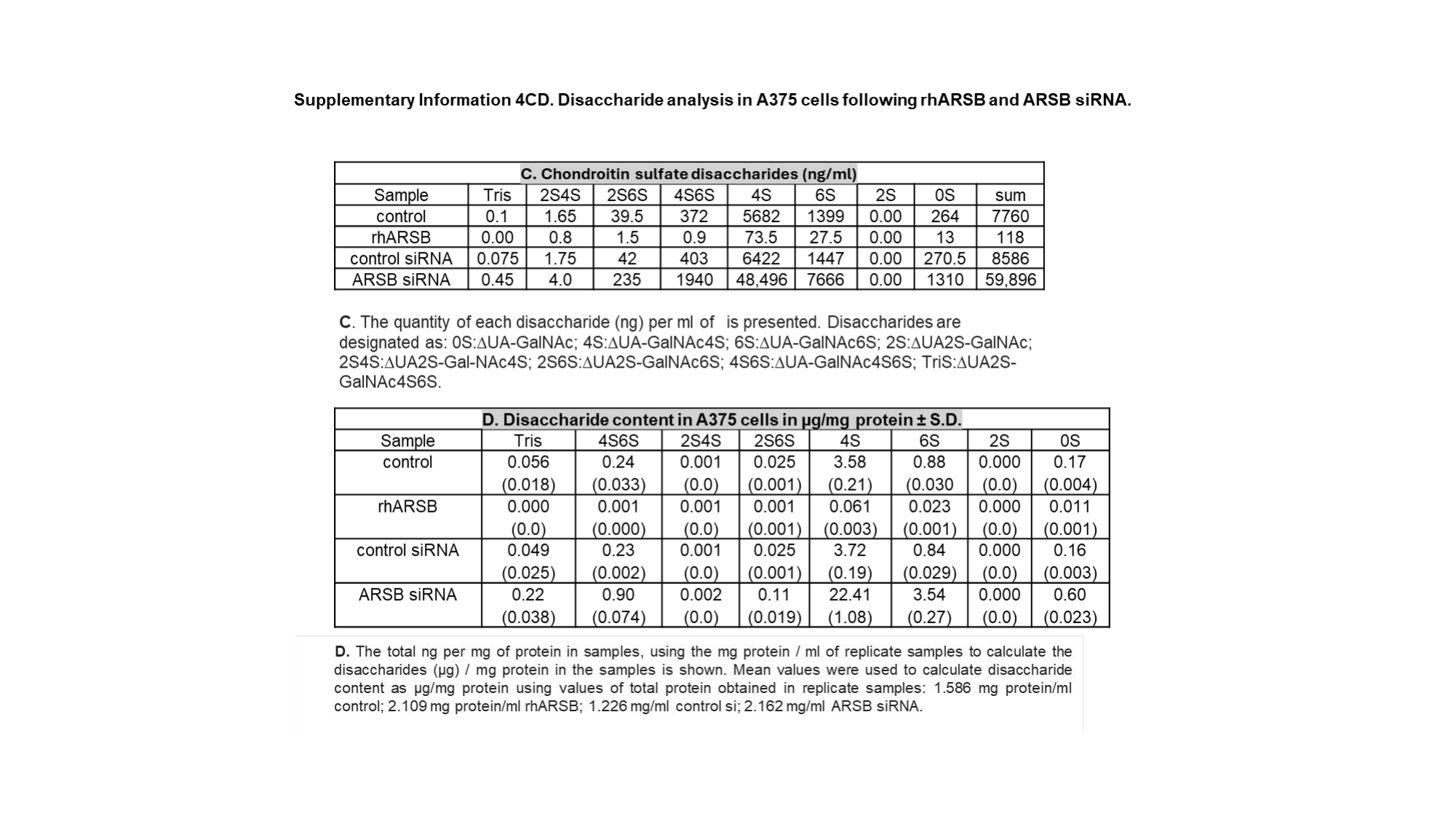

## Slide 8
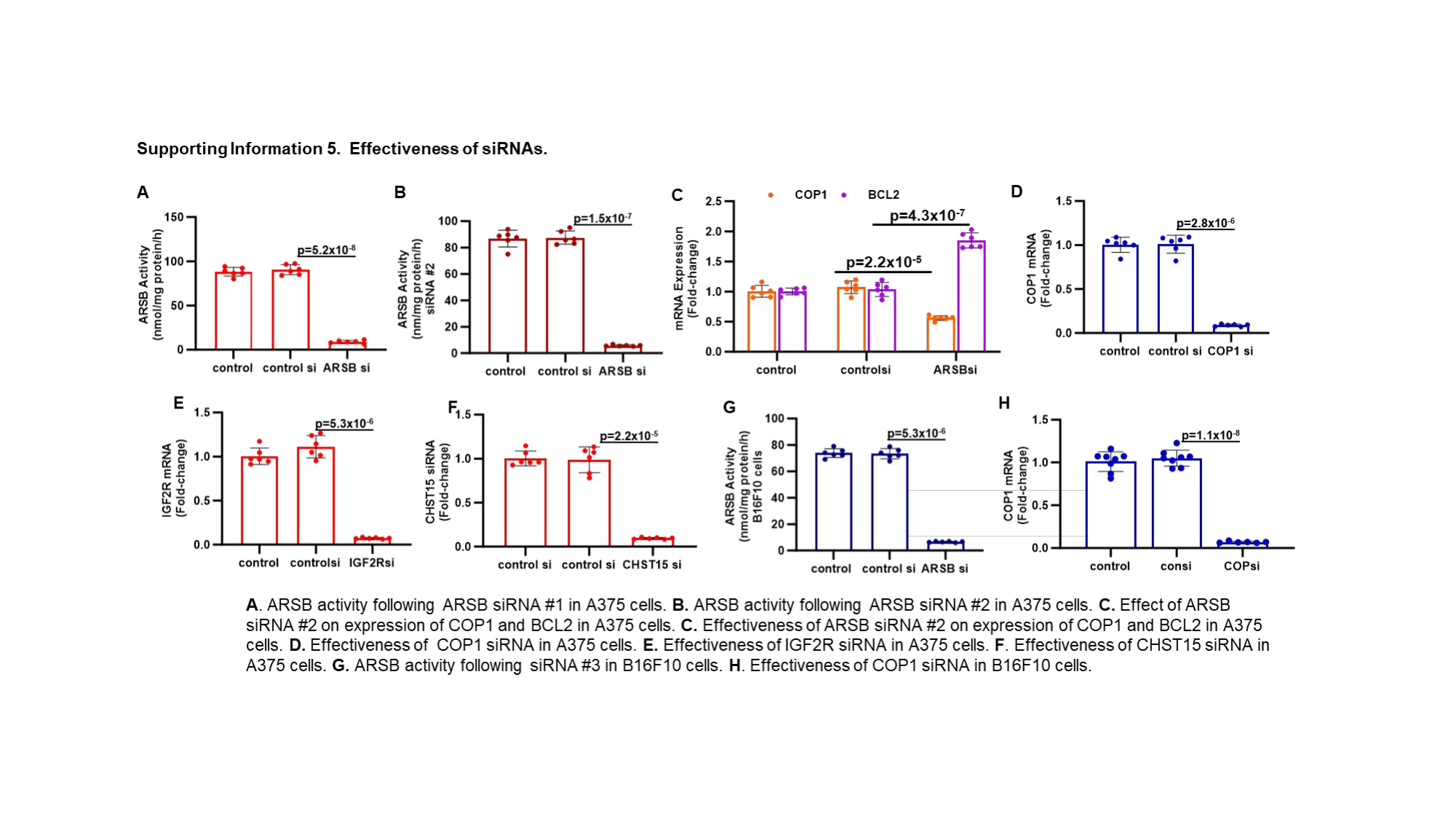

## Slide 9
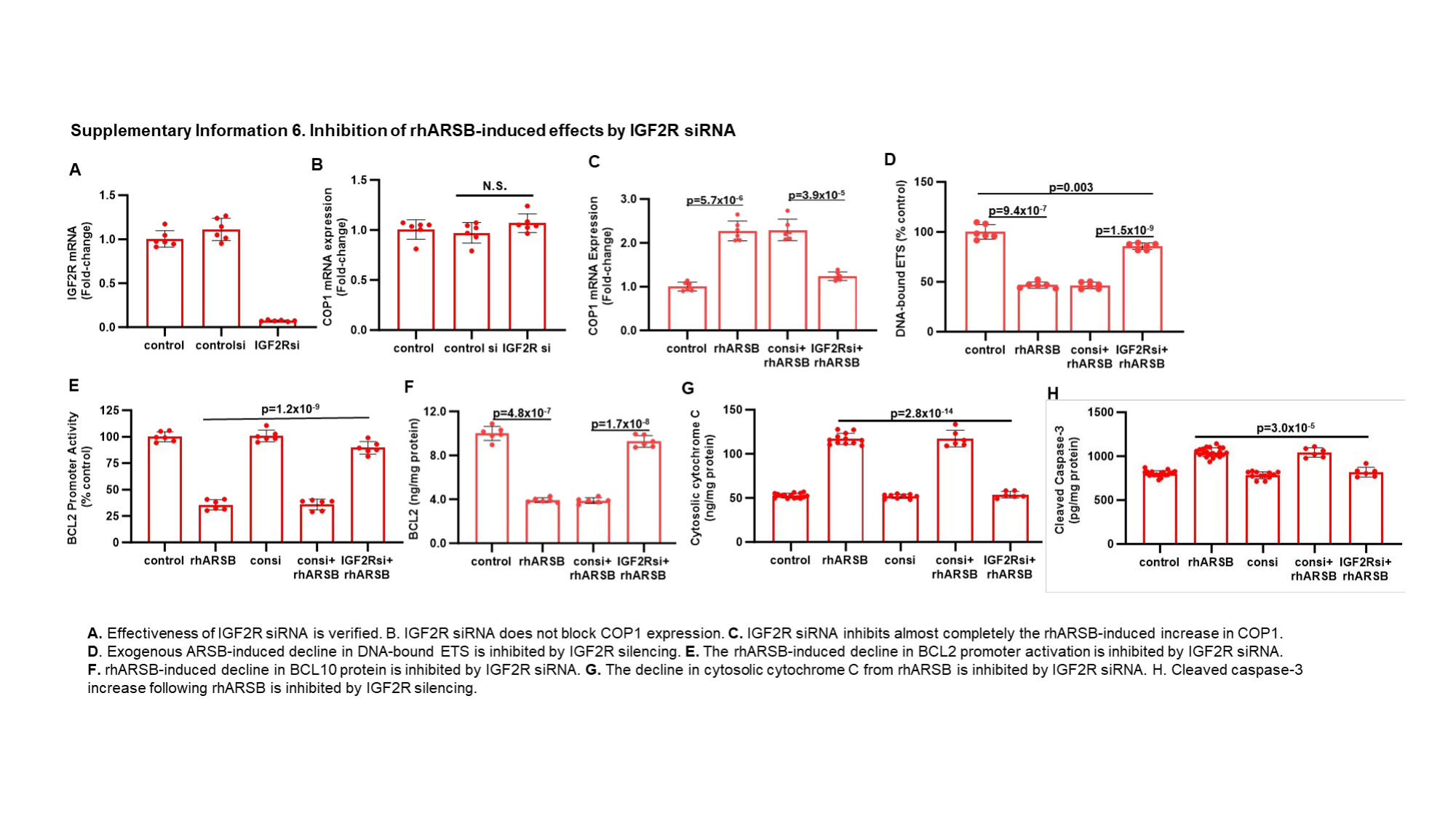

## Slide 10
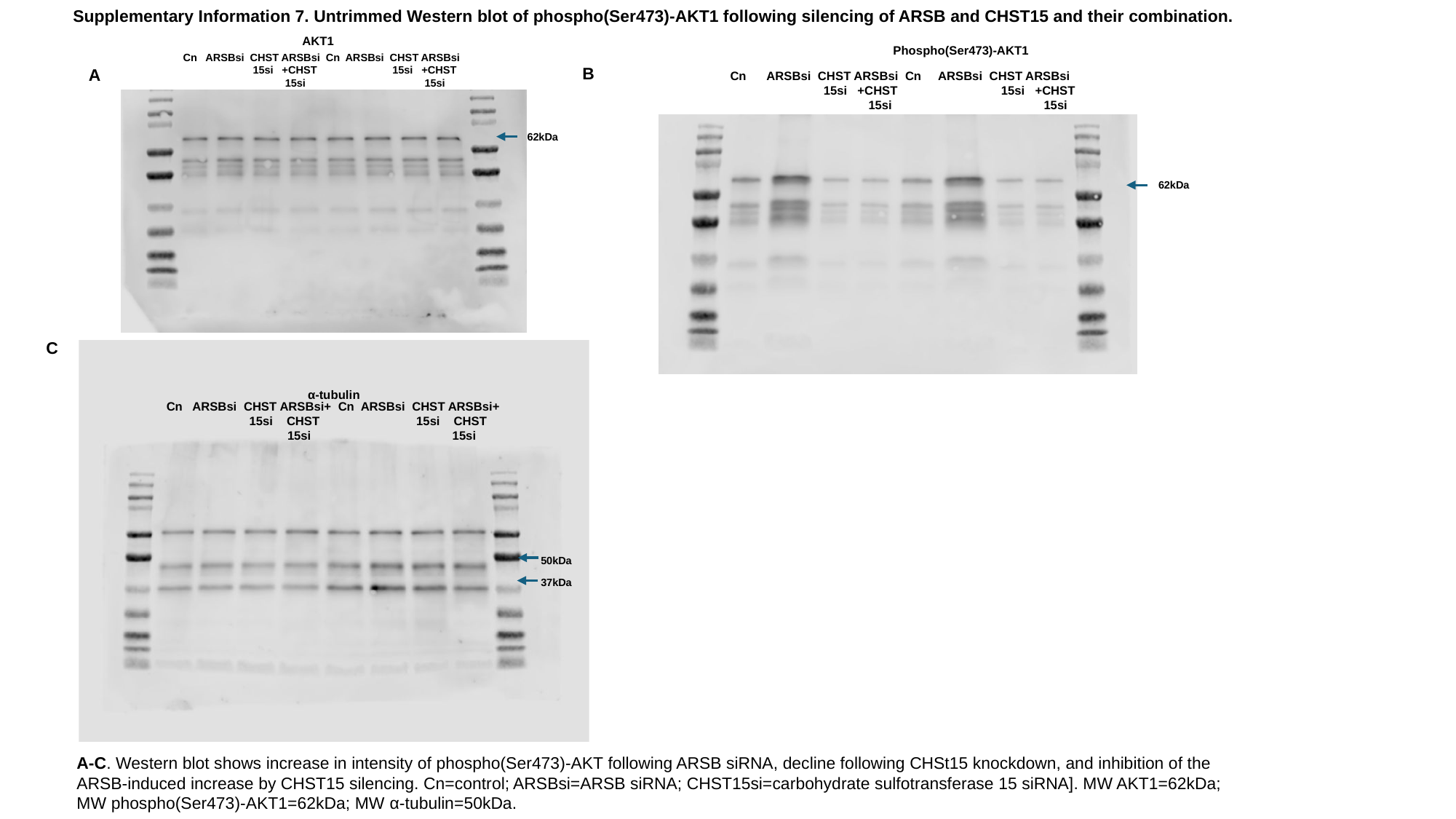

Supplementary Information 7. Untrimmed Western blot of phospho(Ser473)-AKT1 following silencing of ARSB and CHST15 and their combination.
AKT1
α-tubulin
Cn ARSBsi CHST ARSBsi+ Cn ARSBsi CHST ARSBsi+
 15si CHST 15si CHST
 15si 15si
Phospho(Ser473)-AKT1
62kDa
62kDa
50kDa
37kDa
A-C. Western blot shows increase in intensity of phospho(Ser473)-AKT following ARSB siRNA, decline following CHSt15 knockdown, and inhibition of the
ARSB-induced increase by CHST15 silencing. Cn=control; ARSBsi=ARSB siRNA; CHST15si=carbohydrate sulfotransferase 15 siRNA]. MW AKT1=62kDa;
MW phospho(Ser473)-AKT1=62kDa; MW α-tubulin=50kDa.
Cn ARSBsi CHST ARSBsi Cn ARSBsi CHST ARSBsi
 15si +CHST 15si +CHST
 15si 15si
Cn ARSBsi CHST ARSBsi Cn ARSBsi CHST ARSBsi
 15si +CHST 15si +CHST
 15si 15si
B
A
C

## Slide 11
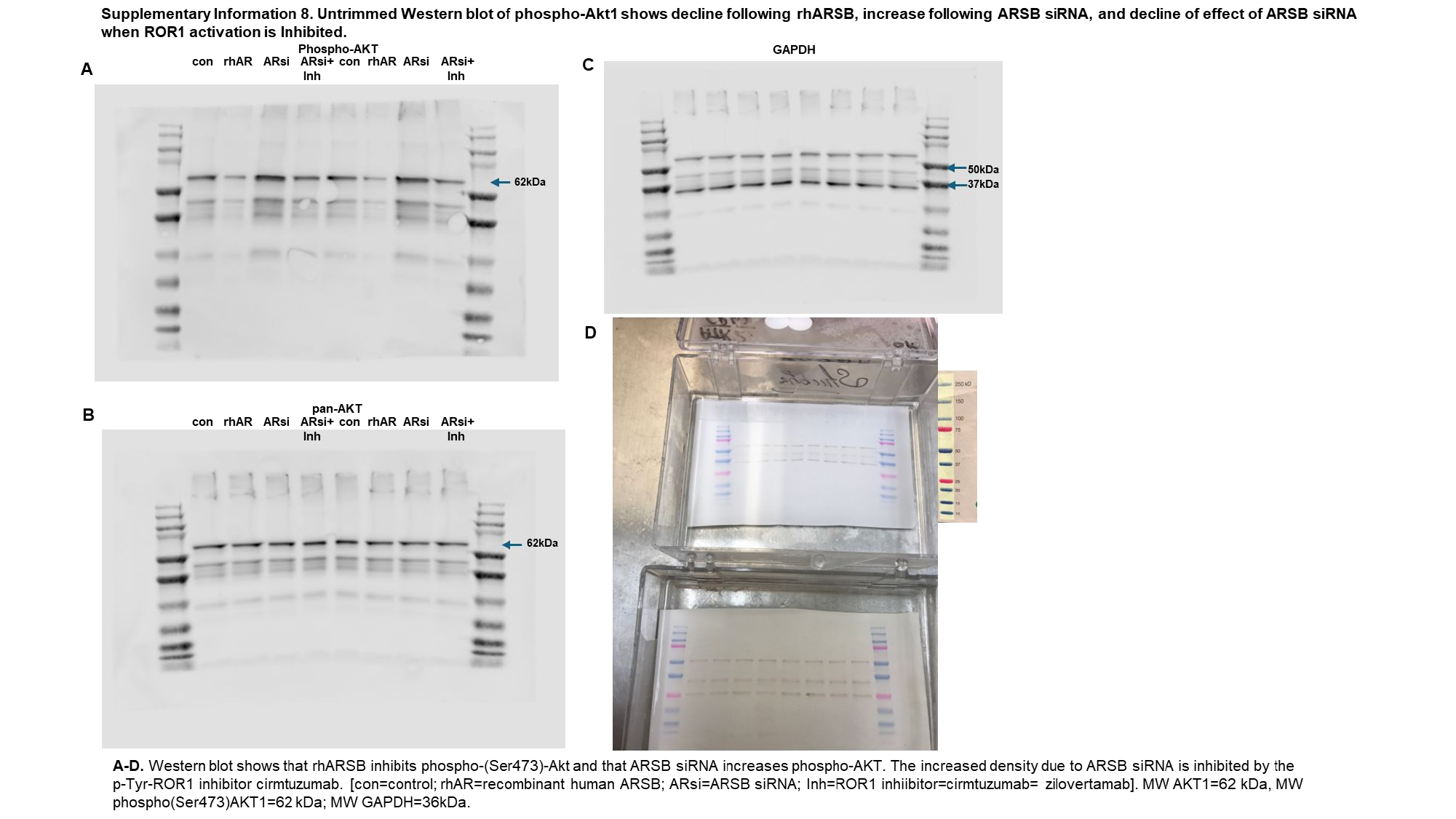

## Slide 12
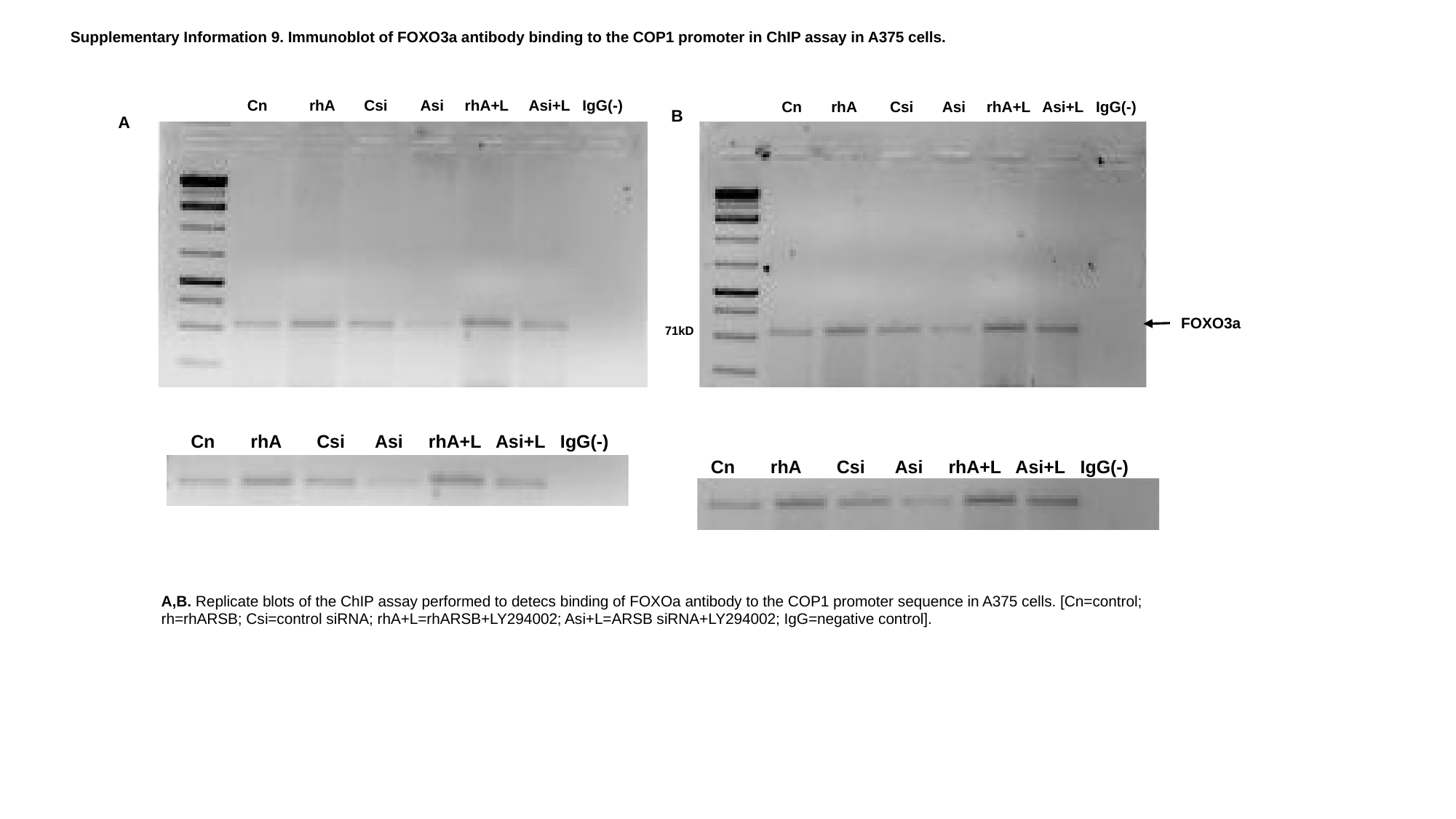

Supplementary Information 9. Immunoblot of FOXO3a antibody binding to the COP1 promoter in ChIP assay in A375 cells.
Cn rhA Csi Asi rhA+L Asi+L IgG(-)
Cn rhA Csi Asi rhA+L Asi+L IgG(-)
 Cn rhA Csi Asi rhA+L Asi+L IgG(-)
FOXO3a
Cn rhA Csi Asi rhA+L Asi+L IgG(-)
A,B. Replicate blots of the ChIP assay performed to detecs binding of FOXOa antibody to the COP1 promoter sequence in A375 cells. [Cn=control; rh=rhARSB; Csi=control siRNA; rhA+L=rhARSB+LY294002; Asi+L=ARSB siRNA+LY294002; IgG=negative control].
B
A
71kD

## Slide 13
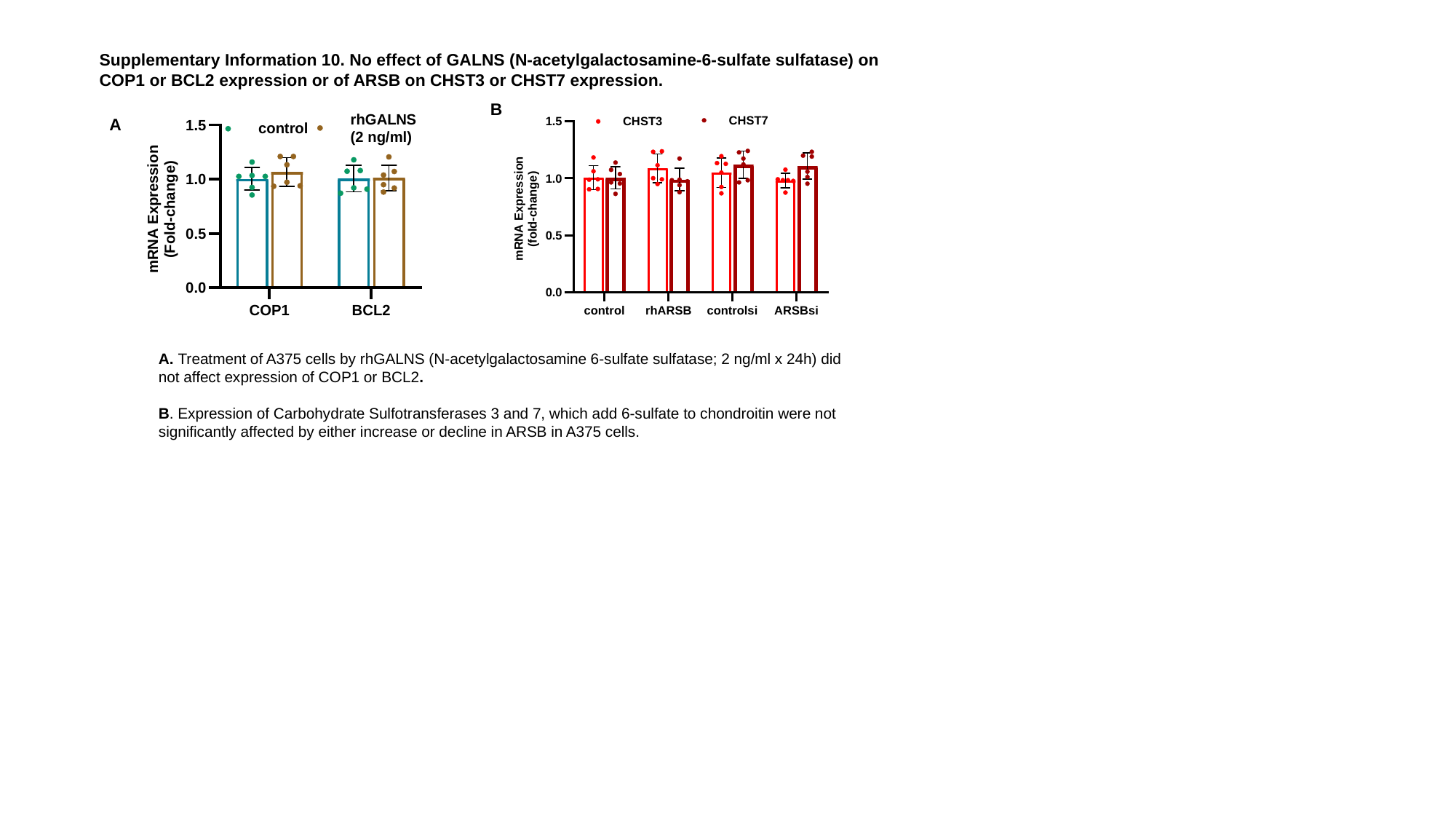

Supplementary Information 10. No effect of GALNS (N-acetylgalactosamine-6-sulfate sulfatase) on
COP1 or BCL2 expression or of ARSB on CHST3 or CHST7 expression.
B
A
A. Treatment of A375 cells by rhGALNS (N-acetylgalactosamine 6-sulfate sulfatase; 2 ng/ml x 24h) did not affect expression of COP1 or BCL2.
B. Expression of Carbohydrate Sulfotransferases 3 and 7, which add 6-sulfate to chondroitin were not significantly affected by either increase or decline in ARSB in A375 cells.
